# Molecular Understanding of the Enhancement in Organic Aerosol Mass at High Relative Humidity

**DOI:** 10.1021/acs.est.2c04587

**Published:** 2023-01-30

**Authors:** Mihnea Surdu, Houssni Lamkaddam, Dongyu S. Wang, David M. Bell, Mao Xiao, Chuan Ping Lee, Dandan Li, Lucía Caudillo, Guillaume Marie, Wiebke Scholz, Mingyi Wang, Brandon Lopez, Ana A. Piedehierro, Farnoush Ataei, Rima Baalbaki, Barbara Bertozzi, Pia Bogert, Zoé Brasseur, Lubna Dada, Jonathan Duplissy, Henning Finkenzeller, Xu-Cheng He, Kristina Höhler, Kimmo Korhonen, Jordan E. Krechmer, Katrianne Lehtipalo, Naser G. A. Mahfouz, Hanna E. Manninen, Ruby Marten, Dario Massabò, Roy Mauldin, Tuukka Petäjä, Joschka Pfeifer, Maxim Philippov, Birte Rörup, Mario Simon, Jiali Shen, Nsikanabasi Silas Umo, Franziska Vogel, Stefan K. Weber, Marcel Zauner-Wieczorek, Rainer Volkamer, Harald Saathoff, Ottmar Möhler, Jasper Kirkby, Douglas R. Worsnop, Markku Kulmala, Frank Stratmann, Armin Hansel, Joachim Curtius, André Welti, Matthieu Riva, Neil M. Donahue, Urs Baltensperger, Imad El Haddad

**Affiliations:** † Laboratory of Atmospheric Chemistry, Paul Scherrer Institute, 5232 Villigen, Switzerland; ‡ Université de Lyon, Université Claude Bernard Lyon 1, CNRS, IRCELYON, 69626 Villeurbanne, France; § Institute for Atmospheric and Environmental Sciences, Goethe University Frankfurt, 60438 Frankfurt am Main, Germany; ∥ Institute for Ion and Applied Physics, University of Innsbruck, 6020 Innsbruck, Austria; ⊥ Division of Chemistry and Chemical Engineering, California Institute of Technology, Pasadena, 91125 California, United States; # Center for Atmospheric Particle Studies, Carnegie Mellon University, 5000 Forbes Avenue, Pittsburgh, 15213 Pennsylvania, United States; ¶ Finnish Meteorological Institute, 00560 Helsinki, Finland; ∇ Department of Experimental Aerosol and Cloud Microphysics, Leibniz Institute for Tropospheric Research, 04318 Leipzig, Germany; ○ Institute for Atmospheric and Earth System Research (INAR)/Physics, Faculty of Science, University of Helsinki, 00014 Helsinki, Finland; ⧫ Institute of Meteorology and Climate Research, Karlsruhe Institute of Technology, 76021 Karlsruhe, Germany; †† Helsinki Institute of Physics, University of Helsinki, 00014 Helsinki, Finland; ‡‡ Department of Chemistry & CIRES, University of Colorado Boulder, UCB 215, Boulder, 80309-0215 Colorado, United States; §§ Department of Applied Physics, University of Eastern Finland, P.O. Box 1627, 70211 Kuopio, Finland; ∥∥ Aerodyne Research, Inc., Billerica, 01821 Massachusetts, United States; ⊥⊥ Atmospheric and Oceanic Sciences, Princeton University, Princeton, 08540 New Jersey, United States; ## CERN, the European Organization for Nuclear Research, CH-1211 Geneva 23, Switzerland; ¶¶ Department of Physics, University of Genoa & INFN, 16146 Genoa, Italy; ∇∇ Department of Chemistry, Carnegie Mellon University, 4400 Fifth Avenue, Pittsburgh, 15213 Pennsylvania, United States; ○○ Department of Atmospheric and Oceanic Sciences, University of Colorado, Boulder, UCB 311, Boulder, 80309 Colorado, United States; ⧫⧫ P. N. Lebedev Physical Institute of the Russian Academy of Sciences, 119991 Moscow, Russia; ††† Tofwerk AG, CH-3600 Thun, Switzerland

**Keywords:** organic aerosol growth, relative humidity, molecular composition, particle water content, particle diffusivity

## Abstract

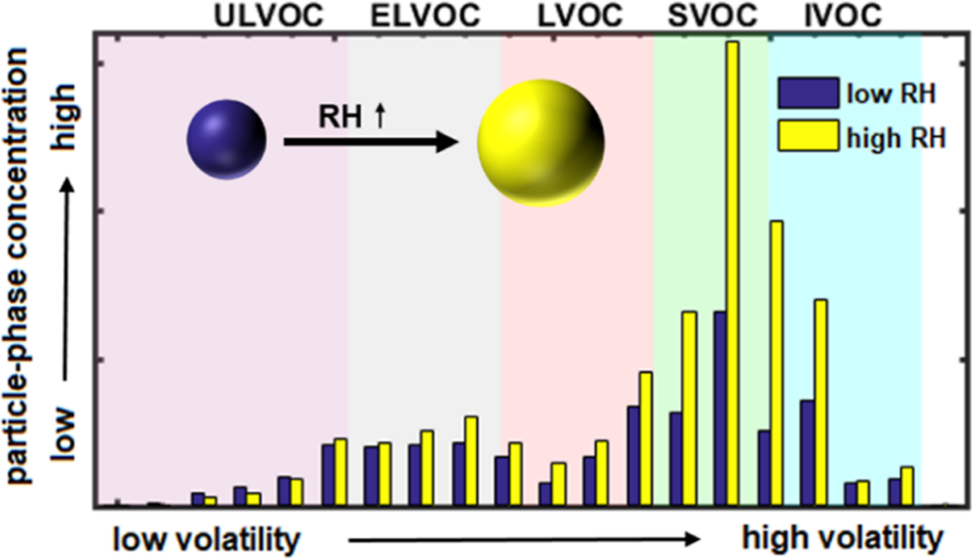

The mechanistic pathway by which high relative humidity (RH) affects gas–particle partitioning remains poorly understood, although many studies report increased secondary organic aerosol (SOA) yields at high RH. Here, we use real-time, molecular measurements of both the gas and particle phase to provide a mechanistic understanding of the effect of RH on the partitioning of biogenic oxidized organic molecules (from α-pinene and isoprene) at low temperatures (243 and 263 K) at the CLOUD chamber at CERN. We observe increases in SOA mass of 45 and 85% with increasing RH from 10–20 to 60–80% at 243 and 263 K, respectively, and attribute it to the increased partitioning of semi-volatile compounds. At 263 K, we measure an increase of a factor 2–4 in the concentration of C_10_H_16_O_2–3_, while the particle-phase concentrations of low-volatility species, such as C_10_H_16_O_6–8_, remain almost constant. This results in a substantial shift in the chemical composition and volatility distribution toward less oxygenated and more volatile species at higher RH (e.g., at 263 K, O/C ratio = 0.55 and 0.40, at RH = 10 and 80%, respectively). By modeling particle growth using an aerosol growth model, which accounts for kinetic limitations, we can explain the enhancement in the semi-volatile fraction through the complementary effect of decreased compound activity and increased bulk-phase diffusivity. Our results highlight the importance of particle water content as a diluting agent and a plasticizer for organic aerosol growth.

## Abstract 2

We report an increase in organic aerosol mass with increasing relative humidity, associated with the increased partitioning of more volatile compounds.

## 1 Introduction

Organic aerosols (OAs) are a ubiquitous and important fraction of submicron atmospheric aerosols, with a large part being secondary organic aerosol (SOA), formed from the oxidation and subsequent condensation of gas-phase precursors.^1^ Understanding the processes affecting the growth and composition of SOA are key steps toward mitigating the environmental effects of atmospheric aerosols.

Water vapor, because of its availability and variability in the atmosphere, can be taken up by particles, affecting the extent of gas–particle partitioning of organic compounds. Previous experimental work has shown mixed effects of relative humidity (RH) on SOA growth, possibly depending on the experimental conditions used and the instrumentation available. Chamber studies studying monoterpene SOA either using high seed concentrations and/or high OA mass (on the order of 100 μg/m^3^) reported no significant changes to SOA mass at high RH.^2,3^ Meanwhile, Prisle et al. (2010)^4^ observed increased monoterpene SOA growth with increasing RH at low initial SOA mass concentration, with this RH effect becoming less significant at higher SOA mass concentrations. Similarly, Pankow et al. (2010)^5^ theoretically predicted an increase of SOA mass with RH, particularly at low SOA loadings where most of the condensable compounds are not already condensed. The increase in SOA mass with RH is experimentally also observed in studies using lower mass concentrations (<20 μg/m^3^).^6,7^ Therefore, this current study focuses on atmospherically relevant mass concentrations (2–4 μg/m^3^ at dry conditions), where the sensitivity to changes in RH should be the highest. Moreover, while previous work was mainly carried out at room temperature, our work aims to replicate free tropospheric conditions through both the low experimental temperatures (243 and 263 K) and relevant mass concentrations.

The RH can affect gas–particle partitioning due to both thermodynamic and kinetic processes. Thermodynamically, the driving force of condensation is given by the difference of the condensable vapor concentration (*C*_g_) and its equilibrium particle-phase concentration (*C*_eq_). Increasing the particle water content would increase the driving force of condensation by decreasing *C*_eq_, effectively diluting the particle and lowering the activity of the organics. Kinetically, condensation may be inhibited at sufficiently low RH, with numerous previous studies reporting uptake limitations due to low particle diffusivity.^7−11^ Low particle diffusivity could lead to higher particle-phase activity at the surface as condensed species are unable to diffuse toward the interior of the particle. At higher RH, particle water can act as a plasticizer, decreasing the viscosity and increasing the particle diffusivity.^12^ Additionally, studies have proposed a reactive uptake of water-soluble organics such as glyoxal and methylglyoxal under high RH conditions, further increasing the SOA mass at high RH.^13,14^ However, some previous work also reported a decrease in SOA mass at high RH for isoprene + NO_*x*_ (nitrogen oxides) and toluene SOA, associated with a decrease in oligomerization at high RH.^15−17^

In this work, through simultaneous online measurements of the molecular composition of the gas and aerosol species, we elucidate the mechanistic pathways by which particle water affects SOA growth from biogenic vapors (α-pinene and isoprene). Experiments have been conducted in a well-controlled environment at the “Cosmics Leaving OUtdoor Droplets” (CLOUD) chamber at CERN (European Organization for Nuclear Research) at low temperatures (243 and 263 K). To explain the observations, we use an aerosol growth model that considers kinetic limitations.

## 2 Materials and Methods

### 2.1 CLOUD Experiment

Results presented here are from the CLOUD14 campaign performed at CERN in the autumn of 2019. The facility is described in more detail elsewhere.^18,19^ Briefly, the CLOUD chamber is a 26.1 m^3^ electropolished stainless-steel chamber with very low background contamination.^20^ Pure air is generated from the evaporation of cryogenic liquid nitrogen and liquid oxygen. The chamber can be temperature-controlled from 208 to 373 K. The RH in the chamber is controlled by flowing a portion of the air through a Nafion humidifier using ultrapure water (18 MΩ cm, Millipore Corporation). Ozone (O_3_) is produced by flowing a small fraction of the air through a quartz tube surrounded by UV-C lights with a wavelength of < 240 nm. Hydroxyl radicals (OH) are formed via O_3_ photolysis using four 200 W Hamamatsu Hg–Xe lamps (wavelength 250–450 nm, adjustable power) throughout the experiments. α-Pinene is introduced to the chamber by passing a small flow of dry air over a temperature-controlled evaporator containing the liquid compound. Isoprene is injected directly from a gas bottle (Carbagas, 0.1% in N_2_). The chamber is run in the continuous-flow mode.

The experiments herein were conducted in the absence of seed aerosol, sulfur dioxide (SO_2_), or NO_*x*_, and experimental conditions are reported in Table S1. Experiments are started by initially stabilizing temperature, RH, and O_3_ in a dark particle-free chamber. Particle formation and growth is then initiated by injecting α-pinene (2–5 ppbV) and isoprene (0–30 ppbV) and turning on the Hg–Xe lamps to generate OH radicals. As steady-state particle growth is achieved, and the RH is gradually increased while keeping all other experimental conditions constant (i.e., oxidation is still ongoing).

A suite of instruments were used to measure the chemical composition of particles and gases sampled from the CLOUD chamber. The particle size distributions were retrieved using a scanning mobility particle sizer (SMPS, size range 9–834 nm, Leibniz Institute for Tropospheric Research, Germany). The O_3_ concentration was measured using a TEI 49C ozone monitor (Thermo Environmental Instruments). The water vapor concentration was measured using a dew point mirror (EdgeTech DewMaster).

### 2.2 Determination of Gas-Phase Species

A proton transfer reaction time-of-flight mass spectrometer was deployed for the measurement of precursor volatile organic compounds (VOCs). Highly oxygenated molecules (HOMs) were measured using a nitrate chemical ionization time-of-flight mass spectrometer (nitrate-CIMS).^21^ The sampling line from the CLOUD chamber to the instrument was actively cooled to the same temperature as that of the CLOUD chamber to avoid any evaporation or transformation of the sampled molecules and clusters. As nitrate ionization is less sensitive toward moderately oxygenated organic compounds (less than six oxygen atoms),^22^ we used an ammonium chemical ionization source coupled to an Orbitrap mass analyzer (hereafter NH_4_^+^–CI-Orbitrap) to measure the full range of gas-phase condensable vapors (Li et al., submitted). NH_3_ was introduced to the ion source from the headspace of a 1% liquid ammonium hydroxide solution (25% NH_3_ basis, ACS reagent, Sigma-Aldrich) at a flow rate of 5 sccm. The analytes were softly charged by binding to ammonium ions, and oxygenated organic compounds down to O_1_ were able to be detected. The NH_4_^+^–CI-Orbitrap data was used as the gas-phase input for the aerosol growth model as it provided non-selective ionization and was therefore able to detect a large majority of the oxidized species.^23^ The gas-phase data from both instruments were corrected using a temperature-dependent sampling-line loss correction factor, as described in Simon et al. (2020).^24^ The gas-phase data are not corrected for wall loss, but the steady-state concentrations are used in the growth model to predict the SOA mass.

### 2.3 Determination of Particle-Phase Species

The particle-phase chemical composition was measured using an extractive electrospray ionization time of flight mass spectrometer (EESI-TOF) and a filter inlet for gases and aerosols coupled to an I^–^ chemical ionization mass spectrometer (FIGAERO-CIMS). The EESI-TOF setup used here is similar to that described in Lopez-Hilfiker et al. (2019).^25^ The electrospray (ES) working solution used was pure water doped with 100 ppm NaI, allowing ions to be detected predominantly as [M + Na]^+^ adducts. The EESI-TOF was applied for sampling for 5 min directly from the CLOUD chamber, followed by 1 min through a HEPA filter (Pall Corporation) for background subtraction. Analyte signals were then converted into the mass flux reaching the detector (ag s^–1^) by scaling with the molecular weight MW_*i*_ of each detected analyte *i* as follows
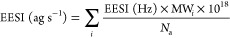
1where *N*_a_ is Avogadro’s number.

The FIGAERO-CIMS is described in detail in Lopez-Hilfiker et al. (2014).^26^ Briefly, particles were collected on a polytetrafluoroethylene filter for 15 min before being heated progressively to be thermally desorbed. The desorbed analytes were detected by iodide (I^–^) chemical ionization.

The particles were also characterized using an aerosol mass spectrometer equipped with a long time-of-flight mass analyzer (AMS-LTOF, Aerodyne Research Inc., USA).

### 2.4 Aerosol Growth Modeling

#### 2.4.1 Base Modeling Run

Aerosol growth is modeled based on the measured gas-phase concentrations of oxidation products and their estimated volatility, as in Surdu et al. (2021).^27^ Compounds measured in the gas phase were distributed in volatility bins according to the volatility basis set framework,^28^ where their volatility was estimated using the parametrization of Stolzenburg et al. (2018),^29^ adapted for α-pinene SOA. Time series of the measured concentrations in each volatility bin were input into the model, together with the condensation sink (CS) measured using the SMPS. The condensation for each volatility bin was therefore simulated at each time step as follows.

The gas-to-particle condensation flux in the *i*th volatility bin, ϕ_*i*_, can be written as
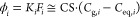
2where *K*_*i*_ is the condensation rate, approximated by the CS, and *F*_*i*_ is the driving force of gas-to-particle partitioning. *C*_g*,i*_ is the measured gas-phase concentration (cm^–3^) and *C*_eq,*i*_ is the equilibrium concentration (cm^–3^) in the *i*th volatility bin. As the mass of particles with a diameter of < 10 nm is negligible, we exclude the Kelvin term (which accounts for the curvature of very small particles) and thus
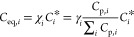
3Here, we assume ideal mixing and use an activity coefficient γ_*i*_ of one as the errors associated with γ_*i*_ are much smaller than the error in estimating volatility (a sensitivity test to volatility is given in Figures S1 and S2). χ_*i*_, *C*_i_^*^, and *C*_p,*i*_ are the activity, saturation vapor concentration (cm^–3^), and modeled particle-phase concentration (cm^–3^) in the *i*th volatility bin, respectively. The CS was calculated as
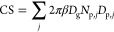
4where  is the correction factor for non-continuum dynamics, *Kn* is the Knudsen number, α is the mass accommodation coefficient (assumed to be one), *D*_g_ is the gas-phase diffusivity (estimated as per Reid et al., 1987),^30^ and *N*_p,*j*_ is the number concentration of particles in the *j*th size bin with diameter *D*_p,*j*_. Thus, the growth of aerosol by all compounds in the *i*th volatility bin can be described as
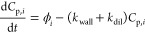
5where *k*_wall_ and *k*_dil_ are the particle wall loss and dilution loss rates (s^–1^), respectively, in the CLOUD chamber. The model is initialized using the measured SMPS volume split equally across all volatility bins *i* in supersaturation.

#### 2.4.2 Gas-Phase Data Input

Data from the NH_4_^+^–CI-Orbitrap were used as the model input, after corrections to reduce humidity-related instrumental variation. The sensitivity of some ions increased with RH; however, most ions’ response remained within a factor of two (Figure S3a). Reasonable correlations to nitrate-CIMS data were obtained for common ions (median *R*^2^ = 0.59, Figure S3).

To reduce the humidity-related variation in the NH_4_^+^–CI-Orbitrap measurements, the steady-state production rate of the gas-phase oxidation products in each volatility bin *i*, prod_ss_,*i*_, was calculated just before the RH ramp and assumed to remain constant during the RH ramp. At steady state, prod_ss_,*i*_ is equal to the total gas-phase loss rate, *K*_loss,*i*_ (cm^–3^ s^–1^)
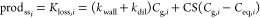
6

The gas-phase concentrations during the RH ramp were then calculated from the steady-state production rate. This provides a great agreement with the measured concentrations from the nitrate-CIMS for common volatility bins (median *R*^2^ = 0.84, Figure S4), validating the assumption that the production rate did not change considerably during the RH ramp. A comparison of the gas-phase time series for different volatility classes is given in Figure S5.

Then, the measured gas-phase signals were scaled using a constant factor, assuming that the ionization efficiency of the NH_4_^+^–CIMS-Orbitrap is the same for all detected species, so that the modeled particle phase volume matches the measured SMPS particle volume at high RH conditions. As particle water was taken into account in the model, the modeled aerosol growth increased and this scaling factor was decreased accordingly.

#### 2.4.3 Consideration of Water Activity

The liquid water content of the aerosol particles was obtained from the estimated hygroscopic growth factor GF(RH) and the measured particle volume, *V*_dry_, from the SMPS. GF(RH) was derived from the hygroscopicity parameter, κ, and the RH, using the following relation^31^
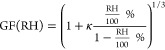
7κ was estimated from the degree of oxygenation of the SOA using the parametrization of Chang et al. (2010)^32^ and κ = 0.29 × (O/C), where O/C was obtained from the AMS for bulk measurements and from the EESI-TOF for O/C determination per volatility bin. Alternatively, κ values were also obtained from the GF parametrization of Massoli et al. (2010),^33^ GF (90% RH) = 0.58 × (O/C) + 0.85. The particle liquid water volume *V*_w_ was then obtained using .

The particle liquid water content was considered in the aerosol growth model by including it in the activity of the organic fraction and modifying eq 3, where *C*_w_ is the particle liquid water concentration (cm^−3^)
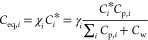
8

We assume that water has little-to-no effect on ideal mixing so that the activity coefficient γ_*i*_ remains one.

#### 2.4.4 Consideration of Bulk-Phase Kinetic Limitations

The kinetic limitation to partitioning was treated using the two-film theory, as described by Zaveri et al. (2014)^34^ and Qin et al. (2021)^7^ in the Model for Simulating Aerosol Interactions and Chemistry (MOSAIC). However, our model is not size-resolved but simulates the condensation of the gas phase across multiple C* bins on a measured CS from SMPS data. This kinetic limitation was introduced by modifying the CS term in eq 4 as follows
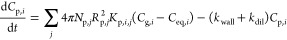
9where *R*_p,*j*_ is the particle radius in the *j*th size bin and *K*_p,*i,*_ is the overall mass transfer coefficient (cm s^–1^) given by Qin et al. (2021)^7^ as
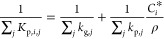
10*k*_g,*j*_ is the gas-side mass transfer coefficient (cm s^–1^) defined as , *k*_p,*j*_ is the particle-side mass transfer coefficient (cm s^–1^) defined as 5*D*_b_/*R*_p,*j*_, *D*_b_ is the bulk diffusivity, and ρ is the molar density [cm^–3^ (particle)] given by *C*_*i*_/*V*_tot_, where *V*_tot_ is the total particle volume.

The bulk diffusivity *D*_b_ is related to viscosity η by the Stokes–Einstein relation
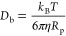
where *k*_B_ is the Boltzmann constant and *T* is the experimental temperature. η was estimated from the glass transition temperature, *T*_g_, using the modified Vogel–Tamman–Fucher equation^35^ and the approach of DeRieux et al. (2018)^36^
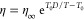
11where η_∞_ is the viscosity at reference temperature (assumed to be 10^–5^ Pa s)^35^ and *T*_0_ is the Vogel temperature, related to *T*_g_ by . *D* is the fragility parameter, assumed to be 10 as in previous studies.^36,37^ Here, the glass transition temperature of the SOA and water mixture, *T*_g_(ω_org_), was used.

The glass transition temperature of the organic species in volatility bin *i* under dry conditions, *T*_g,org,*i*_, was obtained from the molecular weight, MW, and the O/C using the parametrization of Shiraiwa et al. (2017)^37^
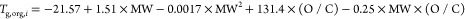
12

The glass transition temperature for the SOA mixture, *T*_g,org_, was then calculated according to the mass fraction ω_*i*_ of each volatility bin *i*: . ω_*i*_ was estimated based on the EESI-TOF measurements. The glass transition temperature of the SOA and water mixture, *T*_g_(ω_org_), was obtained using the Gordon–Taylor equation^38^
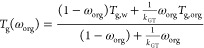
13where ω_org_ is the mass fraction of organics, *T*_g,w_ is the glass transition of pure water (136 K),^12^ and *k*_GT_ is the Gordon–Taylor constant, assumed to be 2.5 for SOA.^38,39^

## 3 Results and Discussion

### 3.1 Molecular-Level Observations during the Humidity Ramp

**Figure 1:**
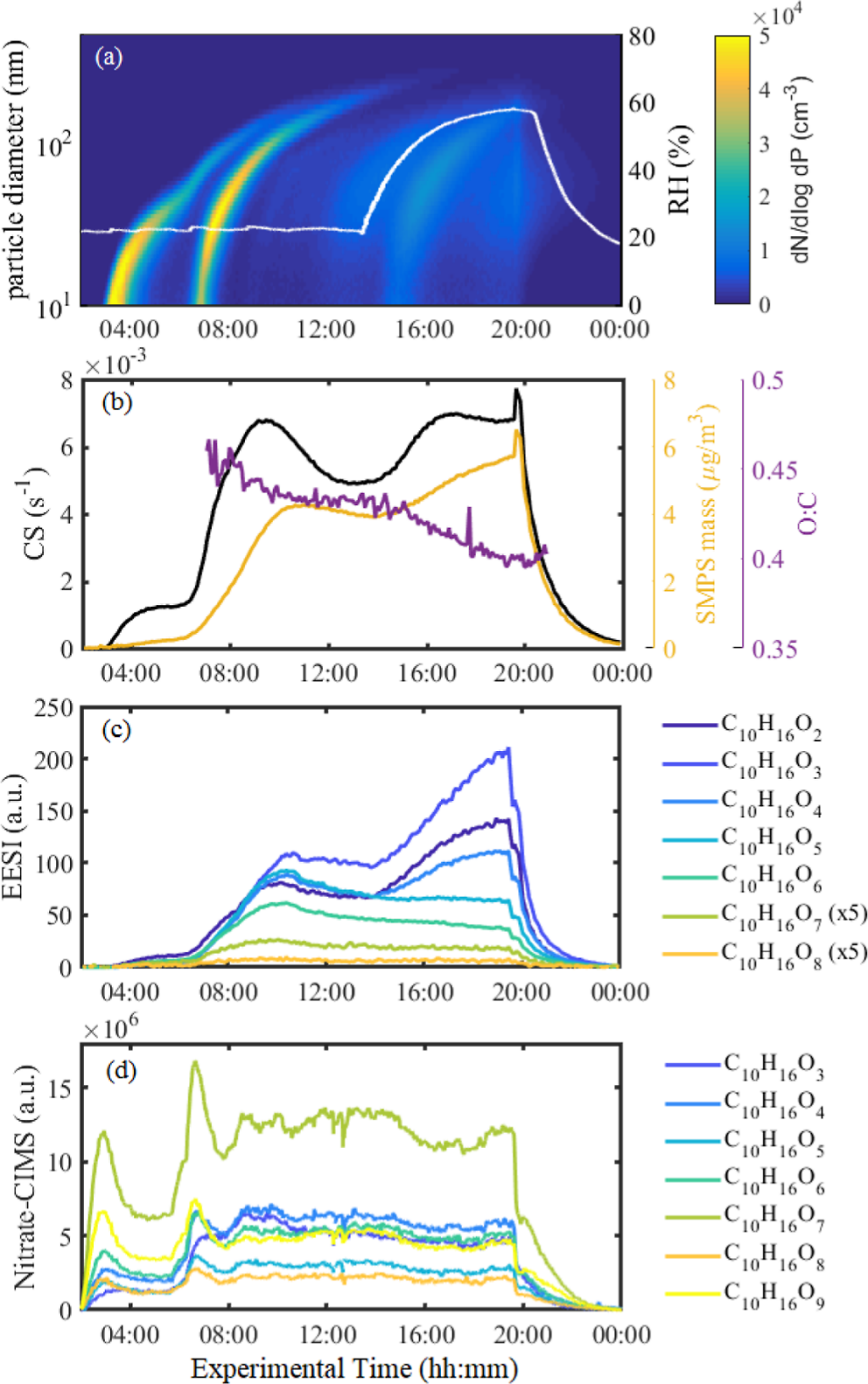
Overview of a pure biogenic oxidation experiment where the RH is ramped up. The experiment is carried out at 243 K. (a) Particle number size distribution from the SMPS and RH trace overlaid in white. (b) Time series of total SMPS mass, CS, and O/C from the AMS. (c) Time series of C_10_H_16_O_2–8_ compounds in the particle phase from the EESI-TOF. C_10_H_16_O_7,8_ signals were multiplied by 5 for better visualization. (d) Time series of C_10_H_16_O_3–9_ compounds in the gas phase from the nitrate-CIMS.

The pure biogenic SOA experiments analyzed in this work are summarized in Table S1, and an overview is given in Figures 1 and S6. The experiment at 243 K (Figure 1) features SOA formation from α-pinene alone, whereas the experiment at 263 K (Figure S6) has a mixture of α-pinene and isoprene. As the RH increases, we observe an increase in the total SOA mass concentration as measured using the SMPS. The SOA mass concentration increases by 45 and 85% as the RH is increased from 10–20 to 60–80% for the experiments at 243 and 263 K, respectively. Since the precursor gas and oxidant concentrations remain unchanged, the increase in mass can be attributed to the RH increase.

Molecular-level data in both the gas and particle phases can be used to investigate which species are responsible for the observed increase in OA mass. In the particle phase, moderately oxygenated compounds C_10_H_16_O_2–5_ increase considerably with increasing RH, whereas highly oxygenated C_10_H_16_O_6–8_ compounds are not affected by the change in RH (Figures 1c, S6c). This results in a measurable change in the bulk O/C at 243 K from 0.44 to 0.40 at 20 and 60% RH, respectively. Likewise, the O/C at 263 K decreases from 0.55 to 0.40 at 10 and 80% RH, respectively. The O/C data from EESI-TOF and AMS show excellent agreement (Figure S7).

In contrast to the particle phase, the entire gaseous C_10_H_16_O_2–8_ compound series behaves consistently as the RH is increased (Figure 1d): we note a decrease in the concentrations of gas-phase species during the RH ramp regardless of their oxygen content. This decrease is consistent with a constant production rate and an increase in the CS. Here, vapors are predominantly lost by condensation to the particles as the CS is larger than the wall loss rate.

**Figure 2:**
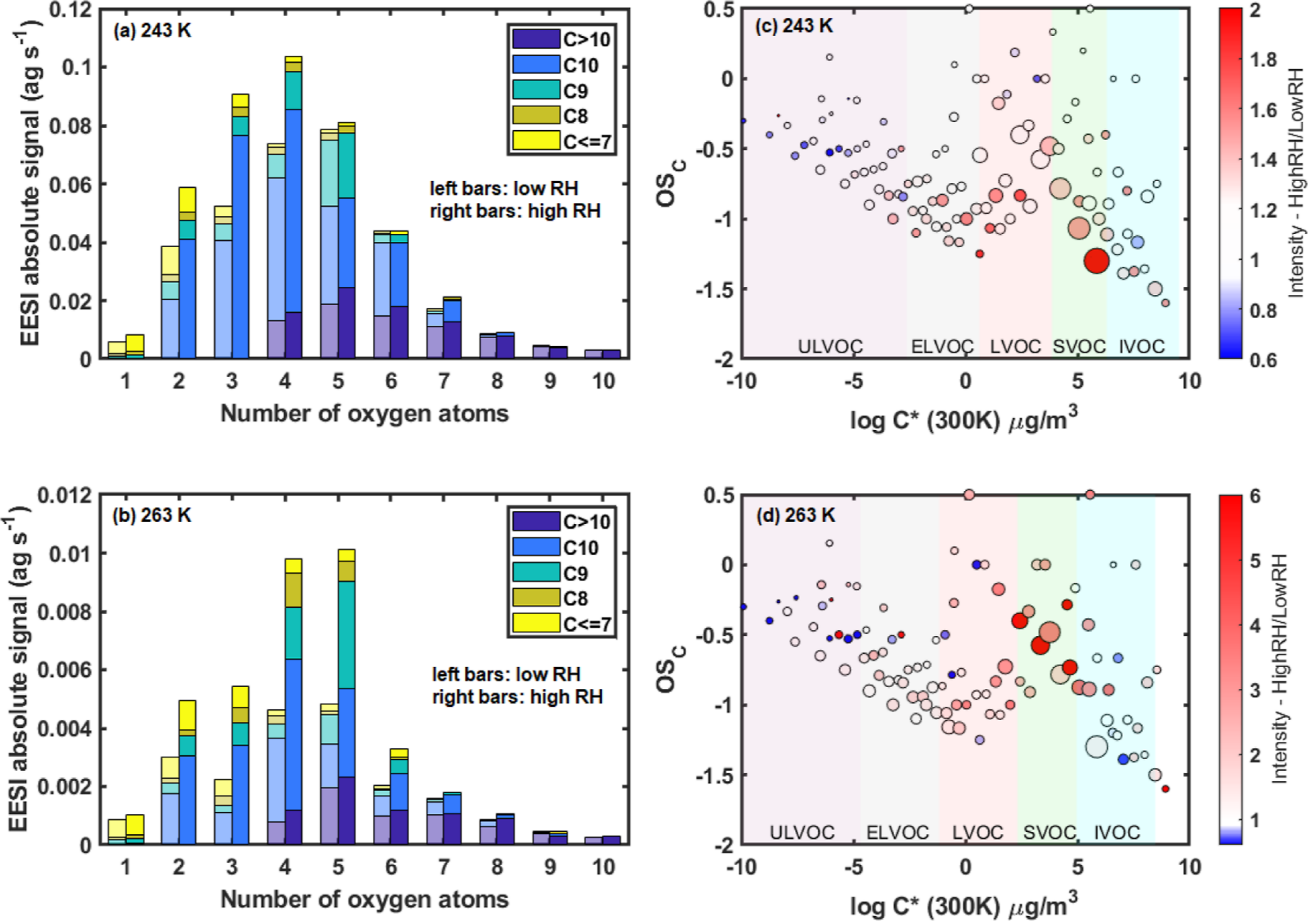
Comparison of the molecular composition of particle-phase compounds at high and low RHs at two different temperatures. (a) Oxygen number histograms at 20 and 60% RH at 243 K, colored by the carbon number. (b) Oxygen number histograms at 10 and 80% RH at 263 K, colored by the carbon number. The bars on the left are for low RH conditions, while the bars on the right represent data at high RH conditions. The RH values shown here represent steady-state low and high RHs for each experiment. Panels (c,d) show the ratios of the carbon oxidation state (OS_C,_ calculated as 2 × O/C – H/C) for high to low RH versus estimated volatility (log C* at 300 K) of particle-phase compounds at 243 and 263 K, respectively. Volatility classes are defined at the experimental temperature and therefore shift with temperature according to the Clausius–Clapeyron relation. Markers are sized by the square root of the ion intensity at high RH, normalized by the largest ion, and colored by the increase in intensity at high RH. Note the difference in the color scales for the ratios for (c,d).

The increase in SOA mass is associated with greater concentrations of less oxygenated higher volatility compounds (mainly O_2_–O_5_), as shown in Figure 2a,b. For example, C_10_H_*x*_O_3_ compounds increase by factors of 1.5 and 3 as the RH is ramped up at 243 and 263 K, respectively. Compounds with carbon numbers C_8_–C_10_ show an increase with RH, while dimers (C_10+_) and smaller compounds (C_<7_) remain largely unchanged during the RH ramp. As shown in Figure 2c,d, most of the mass enhancement at high RH is due to semi- and low-volatility organic compounds (SVOCs and LVOCs, respectively). In contrast, ultra-low- and extremely low-volatility organic compounds (ULVOCs and ELVOCs, respectively) show no increase with RH as they mostly partition to the particle-phase regardless of the presence of water. Intermediate-volatility organic compounds (IVOCs) also remain largely constant at 243 K, while they increase with RH at 263 K. Condensed-phase signals from such species (typically C_<7_) might be due to artifacts in the EESI-TOF, given that they are expected to reside almost entirely in the gas phase. Artifacts may arise due to ionization-induced fragmentation or gas-phase species breaking through the charcoal denuder. Nevertheless, comparison of the EESI-TOF data with the FIGAERO-CIMS data gives excellent time series correlations for common compounds (median *R*^2^ = 0.80, Figure S8) and shows that a similar response to the increase in RH is observed by both instruments.

Multiple reasons may explain the observed effect of RH on the SOA mass concentration and composition, such as water (1) affecting water-dependent gas-phase reaction pathways, (2) increasing OH concentrations from UV photolysis of O_3_, (3) promoting condensed-phase reactions, (4) altering the particle-phase activities of organic compounds or (5) decreasing the particle viscosity. A detailed discussion of these possibilities follows.

First, water vapor could affect the gas-phase chemistry through water reactions with the Criegee intermediates (CIs) produced from ozonolysis. Directly, water vapor could react with the CIs, ultimately forming mainly pinonic acid (C_10_H_16_O_3_). However, as seen in Figure S9, most gas-phase species decrease with the increase in the CS at high RH and we do not see an increase in C_10_H_16_O_3._ We estimate that even at high RH conditions, the reaction rate constant of the CIs with water is at least 2 orders of magnitude lower than that of other Criegee termination pathways, meaning that this pathway is negligible at the low temperatures of this study (Table S2).^40^ Water would also indirectly promote the HO_2_ self-reaction,^41^ reducing related monomers from HO_2_ + RO_2_ reactions and shifting the distribution toward dimers from RO_2_ + RO_2_. However, this pathway is minor at low absolute water concentrations. The reaction rate constant for the HO_2_ + RO_2_ reaction is a factor of six higher than of the HO_2_ self-reaction, even at the highest water concentrations studied here (Table S3).

Water could also increase OH concentrations from O_3_ photolysis, resulting in an increase in C_10_H_18_O_*x*_ compared to C_10_H_14_O_*x*_.^42,43^ However, we observe no changes in the hydrogen distribution of the C_10_ monomers in both the gas and particle phases (Figure S10). Under our low temperature conditions, even at high RH, OH production from α-pinene ozonolysis is factors of 7 and 20 higher than OH from UV photolysis at 243 and 263 K, respectively (Table S4). Overall, our results indicate that changes in gas-phase chemistry with the RH increase have little effect on the distribution of the organic products driving the growth. This is consistent with the results of Li et al. (2019),^3^ who report no changes in the production of HOMs at different RHs, with the majority of HOMs produced from water-independent pathways.

**Figure 3:**
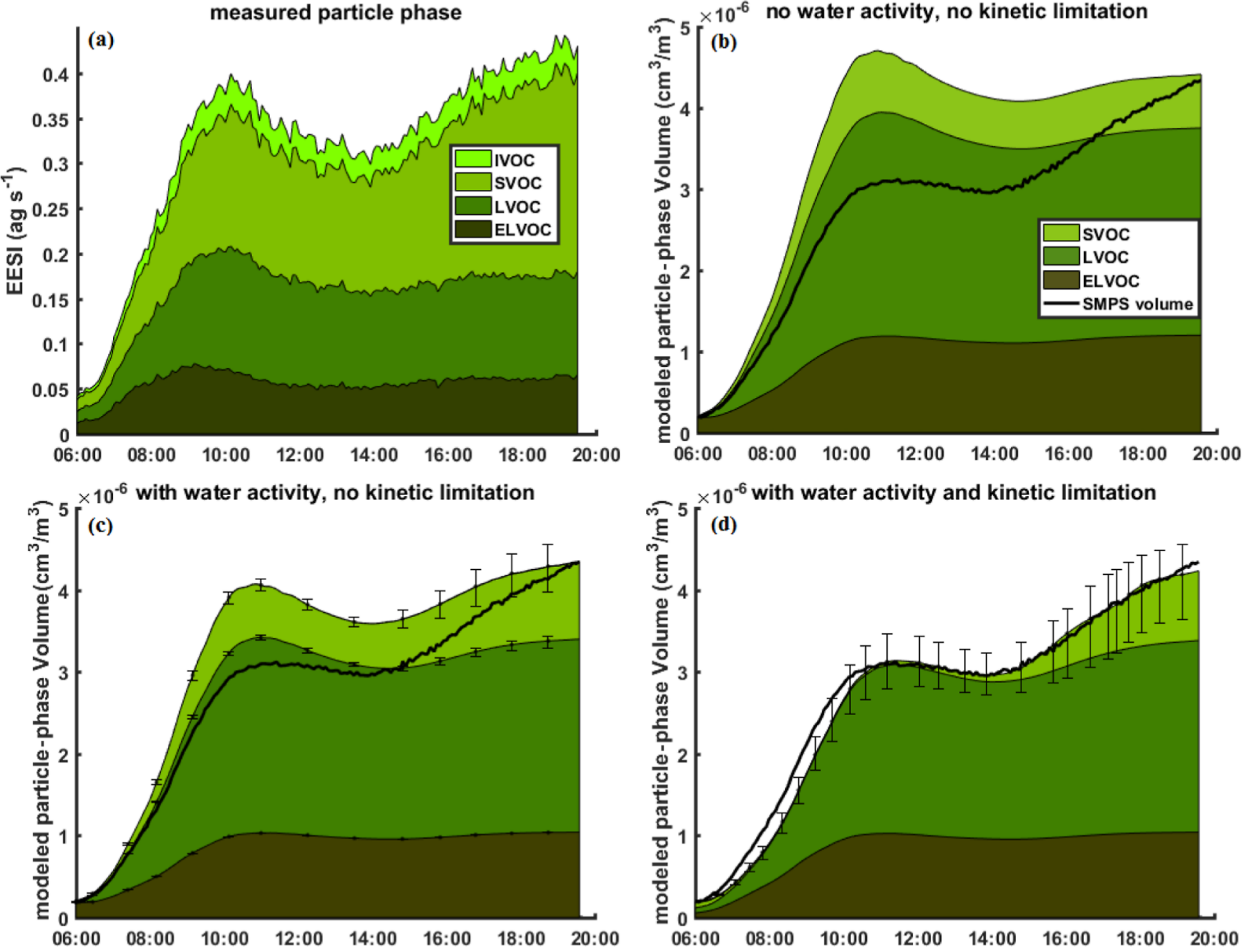
Stacked volumes of ELVOCs, LVOCs, SVOCs, and IVOCs in the particle phase, as measured by the EESI-TOF (a) and predicted by models (b–d) at 243 K. Volatility classes are defined at the experimental temperature. The RH is ramped up continuously from 20% at 14:00 to 60% at 19:45. The model in (b) considers neither water activity nor kinetic limitations to partitioning. The model in (c) just takes into account water activity, and the model in (d) includes both water activity and an easing kinetic limitation with RH. The lower and upper error bars represent the uncertainties from using the hygroscopicity parameter κ as estimated using Massoli et al. (2010) and Chang et al. (2010)., respectively.

Water may also affect condensed-phase chemistry, in this case, possibly leading to the formation of the moderately oxygenated monomer products that we observe to increase at high RH in the particle phase. This would then correspond to a decrease in other classes of molecules as they react away when forming the moderately oxygenated products. Pospisilova et al. (2020) previously observed a rapid decay of C_20_ dimers and some C_10_ species and delayed formation of C_17–18_ and C_7–9_ species.^44^ However, under our experimental conditions, we do not observe this phenomenon in the dimer region as dimer signals remain constant regardless of the carbon number (Figure S11). Similarly, for the monomer region, we observe an increase of both C_10_ and smaller compounds (Figure 2). Unlike Pospisilova et al. (2020), it seems that the SOA enhancement we observe is closely related to the compound’s volatility—under our experimental conditions, only an increase in SVOCs is observed, while more oxygenated LVOCs and ELVOCs remain constant with RH (Figures 3a and S12a).

Finally, the increase in the particle water content at high RH leads to a decrease in the equilibrium concentration *C*_eq_ by decreasing the activity of the organics in the particle phase (eq 8). This would lead to an increase in the driving force of gas–particle partitioning (*C*_g_–*C*_eq_). In addition, water can decrease the particle viscosity and thus increase the bulk diffusivity (*D*_b_), allowing for an enhanced partitioning of semi-volatile species. We model the two effects of particulate water on OA growth below.

### 3.2 Comparison with an Aerosol Growth Model

In Figure 3 (and Figure S12 for the 263 K experiment), we present time series of the volatility distributions during the RH ramp, as measured using the EESI-TOF as well as modeled using an aerosol growth model. At 243 K (Figure 3a), we observe an increase of the SVOC components by 60% as the RH increases from 20 to 60%. Similarly, at 263 K (Figure S12a) the SVOCs increase by 74% and the IVOCs by 88% as the RH increases from 10 to 80%. As mentioned previously, due to denuder breakthrough or fragmentation in the EESI-TOF, it is likely that some of the measured IVOCs are measurement artifacts, leading to an overestimation of the IVOC fraction. At both temperatures, the LVOCs and ELVOCs remain unaffected by the change in RH. We use an aerosol growth model, successively adding the effect of particle water content on decreasing organic activity and increasing the bulk diffusivity to interpret the observations. We note that the model to measurement comparisons are limited by the assumption of uniform sensitivity across all species for both the NH_4_^+^–CI-Orbitrap input data for the model and the EESI-TOF data.

Figure 3b (and Figure S10b for the 263 K experiment) shows the time evolution of the volatility distribution of the base thermodynamic model, neglecting both particle water content and potential kinetic limitations to mass transfer. As in all other modeled experiments, the input gas phase data from the NH_4_^+^–CIMS-Orbitrap is scaled using a constant such that the modeled particle volume matches the observed particle volume at high RH (2nd peak). In this case, the base model overestimates the condensation at low RH, suggesting a lower actual condensation flux. This could be due to diffusive limitations at low RH, which are eased at higher RH. Alternatively, the condensation at high RH could be underestimated as water uptake to the particles could decrease *C*_eq_ by acting as a diluting agent and lowering the activity of the organics.

In Figure 3c (and Figure S10c), we take into consideration the effect of particle water content on the activity of the organic compounds. The particle water content has been estimated based on parametrizations of the hygroscopic growth factor (GF) from the degree of oxygenation of the OA (see the Materials and Methods section, Section 2.4).^32,33^ We estimate the particle water volume for the 243 K experiment to be 1–3.5% of the total volume at 20% RH and 3.5–14% at 60% RH (Figure S13). At 263 K, the particle water volume fraction increases from 0.6–2.4% at 10% RH to 6.5–21% at 80% RH (Figure S13), depending on the parametrization used for the GF. To account for the increase in aerosol growth due to lower organic activities, we decreased the scaling factor for the input gas-phase data in the model, from 3 × 10^7^ to 2.6 × 10^7^, in order to obtain a good agreement at high RH conditions. We show that the consideration of the effect of particle water on the organic species activity provides a better agreement between modeled and measured OA concentrations. Nevertheless, even when considering the highest estimate for the GF,^32^ the model still overestimates the condensation at low RH, suggesting that additional factors inhibit the condensation at low RH.

We include the kinetic limitation to partitioning into our aerosol growth model (also taking into account the particle water content) using the two-film theory, as per Zaveri et al. (2014)^34^ and Qin et al. (2021).^7^ To achieve closure with the observed particle volume, we assume essentially no kinetic limitations at high RH conditions and therefore use the same input *C*_g_ as that in Figure 3c. We find that bulk diffusivity (*D*_b_) values increasing from ∼10^–16^ cm^2^ s^–1^ at low RH to ∼10^–13^ cm^2^ s^–1^ at high RH at 243 K (Figure S14) gives a good agreement between the model and observations, both in terms of the particle volume and compositional change. Similarly, *D*_b_ values increasing from ∼10^–15^ cm^2^ s^–1^ at low RH to ∼10^–12^ cm^2^ s^–1^ at high RH indicated a good agreement at 263 K (Figure S14).

Alternatively, we also compared four commonly used methods of estimating the glass transition temperature (*T*_g_) from the molecular composition (Figure S15).^36,37,45,46^ While the parametrizations agree reasonably well for compounds with volatility higher than LVOCs, they can differ by >100 K for ELVOCs and ULVOCs, leading to differences of up to 40 K in the *T*_g_ of the SOA mixture. Using the parametrized glass transition temperature of Li et al. (2020)^46^ or Zhang et al. (2019),^45^ the *D*_b_ values obtained are unreasonable (less than 10^–50^ cm^2^ s^–1^), prohibiting any condensation. Therefore, we use the estimated *D*_b_ from the *T*_g_ parametrization of Shiraiwa et al. (2017) (see the Materials and Methods section, Section 2.4),^36,37^ obtaining more comparable values of 10^–25^ cm^2^ s^–1^ (low RH) to 10^–19^ cm^2^ s^–1^ (high RH) at 243 K. We note that the Stokes–Einstein relation used to convert between viscosity and *D*_b_ has been shown to break down at high viscosity and can under-predict *D*_b_ by up to 4 orders of magnitude.^47,48^ In particular, strong deviations from Stokes–Einstein behavior are observed at high ratios of glass transition temperature to chamber temperature (*T*_g_/*T*), as is the case here. Sensitivity tests of the model to *D*_b_ are shown in Figure S16 (and Figure S17 for the 263 K experiment), with the *D*_b_ values used in Figure 3d corresponding to decreasing *T*_g_ by ∼40 K. Moreover, due to the complexity of SOA, obtaining accurate values of the Gordon–Taylor mixing constant (*k*_GT_) is a challenge, adding additional uncertainties. In this work, a *k*_GT_ value of 2.5 was used, as in Koop et al. (2011).^38^ However, the values for *k*_*GT*_ for many standards were obtained by Zobrist et al. (2008)^39^ to be in the range of 0.125–5.5 and by Dette et al. (2014)^49^ to be up to 9 for water/MBTCA (SOA–typical acid) mixtures. Given the large errors associated with estimating *D*_b_, especially under the low temperature conditions of this study, we cannot pinpoint the actual *D*_b_ of the particles under consideration. Our results show that *D*_b_ based on parametrizations are lower than that predicted based on aerosol growth, i.e., viscosity plays a smaller role than expected. Nevertheless, the modeling results suggest that changes in organic activity alone are not enough to replicate the observations, and an increase in *D*_b_ by 4 orders of magnitude with RH could provide a possible explanation.

We observe an agreement between the model including both particle water content as well as an easing kinetic limitation (Figures 3d and S12d) and the EESI-TOF data (Figure 3a). In particular, the model predicts accurately the response of different volatility classes to the increase in RH. However, the modeled volatility distribution is considerably different from that measured, namely, IVOCs/SVOCs are more abundant in the measurements, whereas LVOCs dominate the modeled results. The results are likely limited by the assumption of uniform sensitivity in both the NH_4_^+^–CI-Orbitrap gas-phase model input data as well as the EESI-TOF data. The EESI sensitivity is known to vary for different compounds,^50^ and our recent work suggests that EESI sensitivity is higher for more volatile compounds. In addition, the model-to-measurement comparison is also affected by uncertainties in the estimation of the volatility from the molecular formula (sensitivity tests are given in Figure S1) as well as the previously discussed uncertainty in obtaining *D*_b_. Figure S18 shows correlations of time series of modeled and measured ions. On a molecular basis, we note closure between model and measurements for the most abundant species, including C_10_ monomers and moderately oxygenated dimers (O_<8_). Compounds making up 72% of the total EESI signal have a Pearson coefficient of *R* > 0.7 when comparing to the modeled time series. Conversely, poor correlations are observed for small (C_<9_) moderately oxygenated (O_<4_) ions. Such ions make up a considerable portion of the EESI-TOF signal but are predicted to have a low contribution to the particle phase by the model, despite their high concentrations in the gas phase.

### 3.3 Volatility Distributions versus Humidity and Temperature

**Figure 4:**
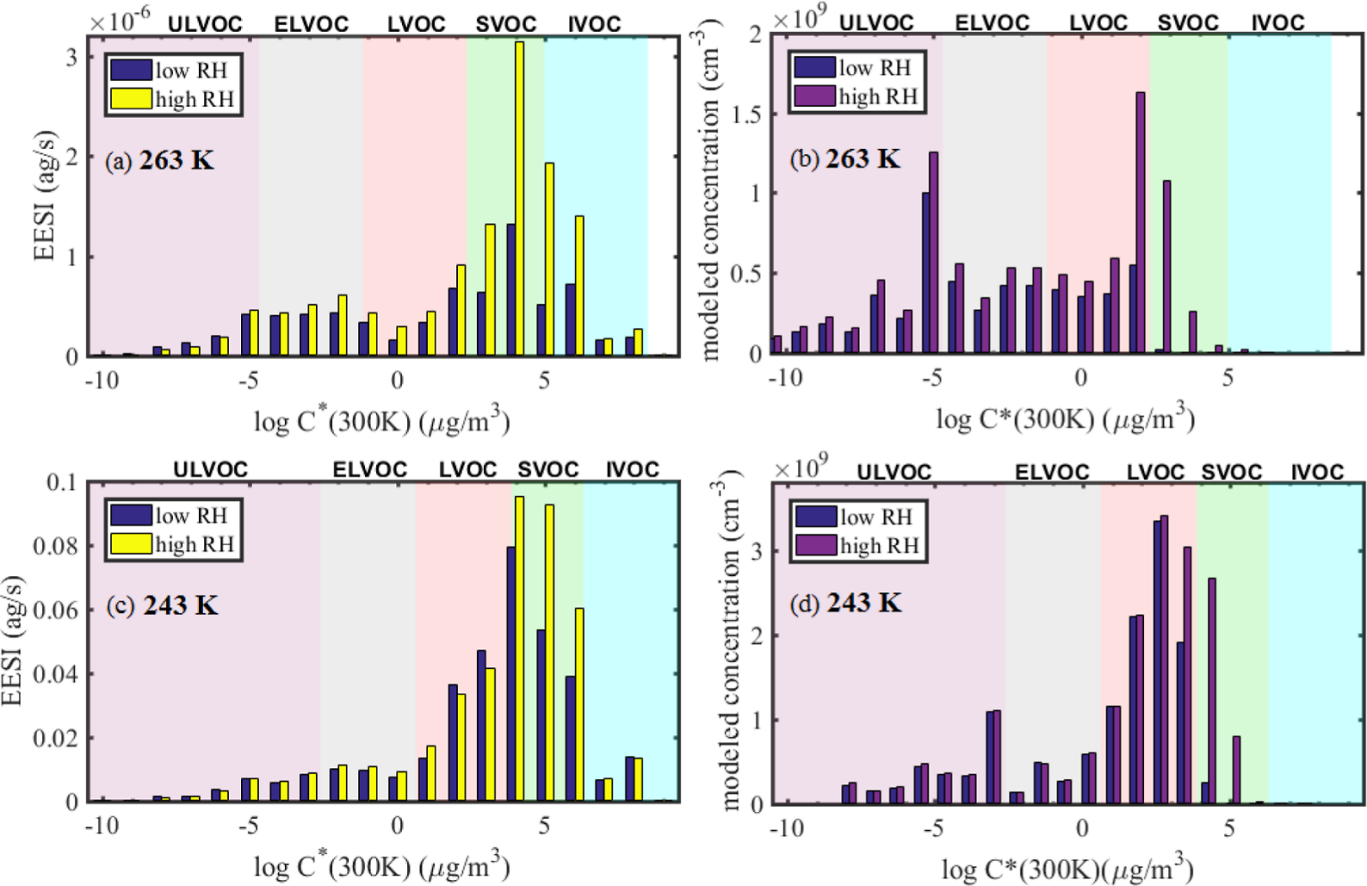
Binned volatility distributions (log C* at 300 K) at low and high RH, as measured by the EESI-TOF (a,c) and modeled (b,d). Panels (a,b) show data at 263 K, while panels (c,d) show data at 243 K. Model data are from the model including both the particle water content and an easing kinetic limitation, as in Figure 3d. Volatility classes are defined at the experimental temperature and therefore shift with temperature according to the Clausius–Clapeyron relation.

Binned volatility distributions of experimental and modeled particle phase for experiments at 263 K (α-pinene and isoprene) and 243 K (α-pinene only) are given in Figure 4, for both low RH and high RH conditions. The volatility distributions feature a main peak in the LVOC–SVOC range, corresponding mainly to monomer (C_10_) products, with a secondary peak in the ELVOC–ULVOC range, associated with dimer (C_20_) products. Recently, the effect of the chemical composition of adding isoprene to α-pinene nucleation and growth was analyzed for the experiment at 243 K in this study.^51^ The authors found no significant differences in the volatility distribution from the addition of isoprene compared to the addition of pure α-pinene, with large differences in volatility distributions in experiments at different temperatures. Here, we plot the intrinsic volatility (i.e., C* at 300 K) on the *x*-axis, while volatility classes are defined at the experimental temperature and shifted according to the Clausius–Clapeyron relation. Therefore, the saturation vapor pressure of the measured compounds decreases with temperature, and compounds with higher intrinsic volatility are able to contribute to particle growth. This is seen in the EESI data as the bins with log_10_C* (300 K) = 5–6 contribute 17% of the total signal at 263 K and 27% at 243 K, at low RH conditions. In contrast, at higher temperatures, HOM formation through autoxidation is increased, producing species with lower intrinsic volatility.^24^ We observe this as a higher fraction of compounds with intrinsically lower volatility for the 263 K experiment than in the 243 K experiment, both in the measurements and model.

According to the model, the SVOC fraction is estimated to increase from 3 to 25% at 243 K and from 1 to 17% at 263 K, at low and high RHs, respectively. Similarly, the measured SVOC fraction increases from 40 to 51% at 243 K and 34 to 47% at 263 K, at low and high RHs, respectively. A marked shift of the chemical composition to higher volatility species leading to an overall increase in mass is seen both in the measurements and the model results. We find that at both temperatures, the particle phase concentrations of species with very low volatility (ULVOCs, ELVOCs, and most LVOCs) is not enhanced at high RH. Species with moderate volatilities (SVOCs, some LVOCs, and some IVOCs) see a significant increase at high RH, whereas more volatile species (IVOCs) do not contribute much to the particle phase, regardless of RH. Kinetically, the limitation due to low *D*_b_ does not apply for species with sufficiently low C* as they are able to condense regardless of the activity at the particle surface. In contrast, for too large values of C*, condensation is not favored and these species reside in the gas phase.

We expect that with increasing temperature, the kinetic limitation to partitioning will be diminished, and therefore, the extent of the OA enhancement will decrease with temperature. However, depending on the type of OA and hence the particle phase state, kinetic limitations may still apply at room temperature. Thermodynamically, at higher temperatures, particle water could also lower organic activities by acting as a diluting agent, enhancing the OA uptake. This is in agreement with previous room temperature monoterpene SOA studies, which observe an OA enhancement at high RH.^6,7^ The two experiments in this study are likely to have different OA mass enhancements (45% under 243 K and 85% under 263 K) due to the varying initial (low RH) mass concentrations (∼4 and 2 μg/m^3^, respectively), given the high sensitivity of the RH effect to OA mass.^5^ Therefore, this study provides a molecular understanding of OA enhancement with increasing RH under atmospherically relevant conditions, rather than deriving a general dependence of the OA enhancement with RH at different temperatures.

### 3.4 Implications

We report an increase in SOA growth with increasing RH. Using simultaneous real-time and molecular measurements of the gas- and particle-phase composition, we are able to pinpoint the increase in mass to the increased condensation of semi-volatile species. This was previously a challenge, mainly due to limitations in molecular-level particle-phase measurement techniques, with former studies either resorting to offline filter-based measurements or unable to track changes in the chemical composition.^2,15^ The low detection limits of the EESI-TOF and a well-controlled environment at CLOUD allow for observations at atmospherically relevant mass concentrations. This work is therefore the first experimental demonstration of the theoretical framework of Pankow (2010), predicting that the effect of RH on condensation would be the largest at low mass concentrations, where large fractions of condensable compounds are not already condensed. At both 263 and 243 K, we are able to explain the observations by considering the complementary effect of particle water on decreased particle activities and increased bulk diffusion. We show that at free tropospheric temperatures, we have to invoke a kinetic limitation to partitioning under dry conditions. At boundary layer temperatures, however, we expect the particles to be less viscous, and thus, partitioning would be less affected by the particle water content. Future studies of aerosol growth should investigate different types of OA (e.g., urban and marine) as well as different aerosol mixing states. Under our experimental conditions (e.g., 263 K and an OA mass concentration of ∼ 3 μg/m^3^), we observe around a factor of 2 increase in mass as the RH is increased from 10 to 80%. This strong effect of RH on partitioning can be included in applied regional models investigating the free troposphere, where temperature and mass concentrations are similar, to capture SOA enhancement. Such levels of particle water content could further enhance SOA through condensed-phase reactions. Although we were not able to detect these reactions due to the low chamber lifetime (∼105 min), this should be investigated in future work.

## Supporting Information Available

Details of experimental conditions; reaction rate constants pathways affected by water; volatility sensitivity tests of the growth model; validation of gas-phase model data input; overview of the experiment at 263 K; comparison of O/C from different methods; comparison of EESI and FIGAERO data; comparison of the gas-phase chemical composition at low and high RHs; model results for the experiment at 263 K; estimated particle-phase water volume; comparison of estimated bulk diffusivity; model sensitivity tests to bulk diffusivity; and molecular comparison of the model and measurements (PDF)
