# Supplementary material for: Molecular Understanding of the Enhancement in Organic Aerosol Mass at High Relative Humidity
Source: Environ Sci Technol. 2023 Jan 30;57(6):2297–309. doi: 10.1021/acs.est.2c04587 (PMC9933880; doi:10.1021/acs.est.2c04587)
Supplement: Supplementary file 1 — es2c04587_si_001.pdf [file es2c04587_si_001.pdf]

Supporting Information for

Molecular understanding of the enhancement in organic aerosol mass at high relative humidity

Mihnea Surdu[1], Houssni Lamkaddam[1], Dongyu S. Wang[1], David M. Bell[1], Mao Xiao[1], Chuan Ping Lee[1], Dandan Li[2], Lucía Caudillo[3], Guillaume Marie[3], Wiebke Scholz[4], Mingyi Wang[5,6], Brandon Lopez[6], Ana A. Piedehierro[7], Farnoush Ataei[8], Rima Baalbaki[9], Barbara Bertozzi[10], Pia Bogert[10], Zoé Brasseur[9], Lubna Dada[1], Jonathan Duplissy[10,11], Henning Finkenzeller[12], Xu-Cheng He[9], Kristina Höhler[10], Kimmo Korhonen[13], Jordan E. Krechmer[14], Katrianne Lehtipalo[7,9], Naser G.A. Mahfouz[15], Hanna E. Manninen[16], Ruby Marten[1], Dario Massabò[17], Roy Mauldin[18,19], Tuukka Petäjä[9], Joschka Pfeifer[16], Maxim Philippov[20], Birte Rörup[9], Mario Simon[3], Jiali Shen[9], Nsikanabasi Silas Umo[10], Franziska Vogel[10], Stefan K. Weber[3,16], Marcel Zauner-Wieczorek[3], Rainer Volkamer[12], Harald Saathoff[10], Ottmar Möhler[10], Jasper Kirkby[16], Douglas R. Worsnop [9,14], Markku Kulmala[9], Frank Stratmann[8], Armin Hansel[4], Joachim Curtius[3], André Welti[7], Matthieu Riva[2,21], Neil M. Donahue[6], Urs Baltensperger[1], Imad El Haddad[1]*

[*]Corresponding author; email: imad.el-haddad@psi.ch

This file contains:

Pages S1 to S16

Tables S1 to S4

Figures S1 to S18

References (1 to 11)

Supplementary Table 1. List of experimental conditions for the CLOUD 14 experiments analyzed in this work. Both experiments were carried out in the absence of NO_x and SO_2. Both experiments also included a gradual increase in RH. Alpha-pinene (AP) and isoprene (IP) values refer to steady-state mixing ratios.

**Experiment number**	**Temperature (K)**	**RH (%)**	**Peak mass concentration (μg/m^3)**	**AP (ppbV)**	**IP (ppbV)**	**Ozone (ppbV)**
**1**	243	20 to 60	5.7	5	30	100
**2**	263	10 to 80	3	2	0	110

Supplementary Table 2. Reaction rate constants for unimolecular reaction (k_u) and bimolecular reaction (k_b) of the Criegee intermediates, calculated based on acetone oxide data from Long et al. (2018). Data is given for the experimental conditions presented in Table S1.

**Temperature (K)**	**k_u (s)**	**$[H_2O]_{low\ RH}$ (molecules cm^{-3})**	**$[H_2O]\cdot k_{b,\ low\ RH}$ (s^{-1})**	**$[H_2O]_{high\ RH}$ (molecules cm^{-3})**	**$[H_2O]\cdot k_{b,\ high\ RH}$ (s^{-1})**
243	40.3	1.15e15	5.05e-3	3.44e15	1.51e-2
263	65.7	3.93e15	2.39e-2	3.15e16	1.91e-1

Supplementary Table 3. Reaction rate constants for HO_2+RO_2 reaction ($k_{HO2+RO2}$) and HO_2 self-reaction in the presence of water ($k_{HO2+HO2}$), obtained from Master Chemical Mechanism, MCM v3.3.1 (Jenkin et al, 1997; Saunders et al, 2003), via website: http://mcm.leeds.ac.uk/MCM and Stone and Rowley (2005), respectively. Data is given for the experimental conditions presented in Table S1.

**Temperature (K)**	**$k_{HO2+RO2}$**	**$k_{HO2+HO2-lowRH}$**	**$k_{HO2+HO2-highRH}$**
243	6.13e-11	9.07e-12	9.95e-12
263	4.08e-11	5.62e-12	7.15e-12

Supplementary Table 4. Comparison of OH production from photolysis, at low and high RH, and from α-pinene ozonolysis. The photolysis rate coefficient of O_3 to O^1D + O_2 by the UV lamps was experimentally determined to be 5.7e-5 s^{-1}. Reaction rate constants for the reactions of O^1D were obtained from MCM (Jenkin et al, 1997; Saunders et al, 2003). The OH formation yield from α-pinene ozonolysis was taken to be 0.85 at both temperatures (Atkinson et al., 1992). Data is given for the experimental conditions presented in Table S1.

**Temperature (K)**	**$[OH]_{photolysis,\ low\ RH}$ (molecules cm^{-3})**	**$[OH]_{photolysis,\ high\ RH}$ (molecules cm^{-3})**	**$[OH]_{AP}$ (molecules cm^{-3})**
243	3.84e4	1.15e5	1.49e7
263	1.35e5	1.08e6	7.27e6

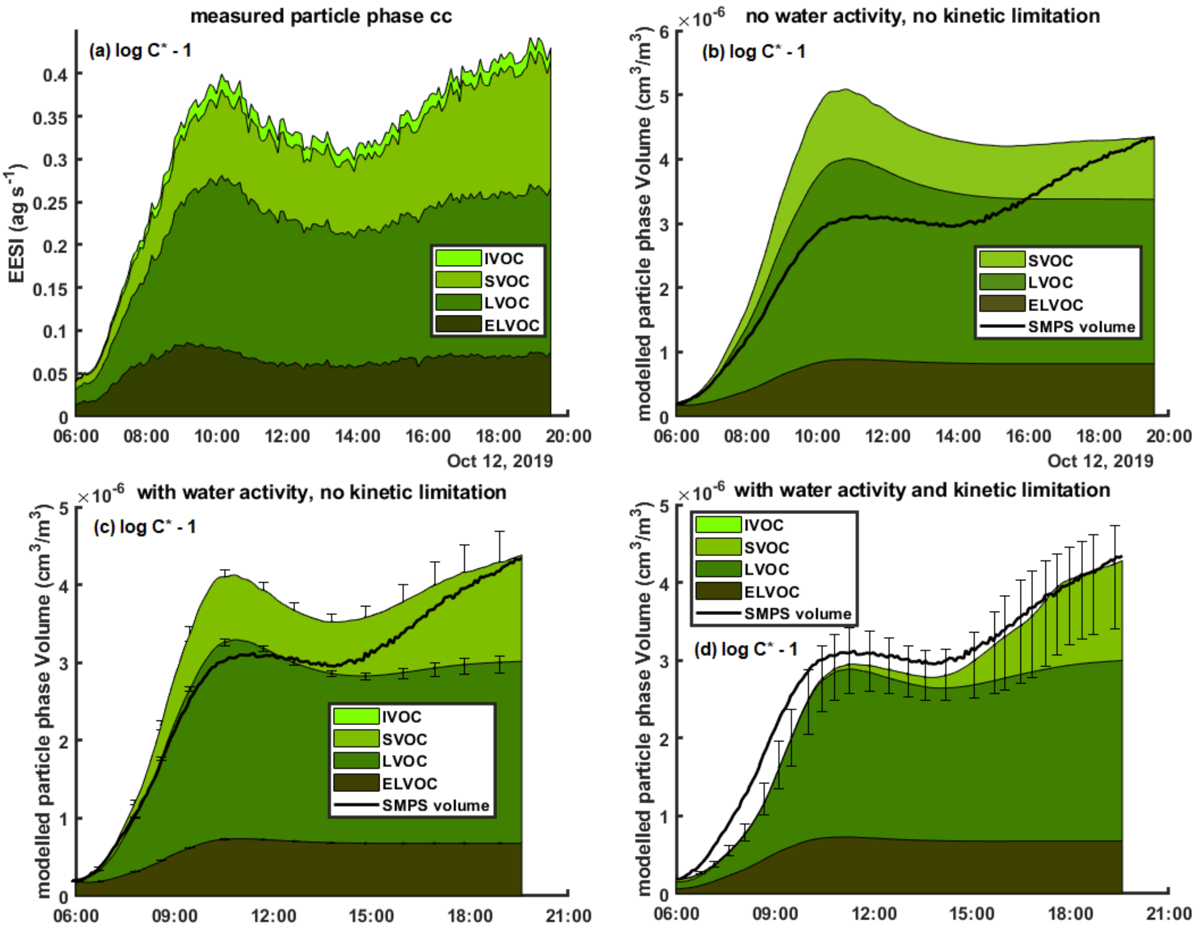

Supplementary Figure 1. Volatility sensitivity test of aerosol growth model with reducing log *C** by 1, at 243 K. Stacked signals of ELVOC, LVOC, SVOC and IVOCs in the particle phase, as measured by the EESI-TOF **(a)** and volumes measured by the SMPS and predicted by the different models **(b-d)**. Volatility classes are defined at the experimental temperature. The RH is ramped up continuously from 20% at 14:00 to 60% at 19:45. The model in **(b)** takes into account neither water activity nor kinetic limitations to partitioning. The model in **(c)** just takes into account water activity and the model in **(d)** includes both water activity and an easing kinetic limitation with RH. The lower and upper error bars are based on using the growth factor as estimated using Massoli et al. (2010) and Chang et al. (2010), respectively.

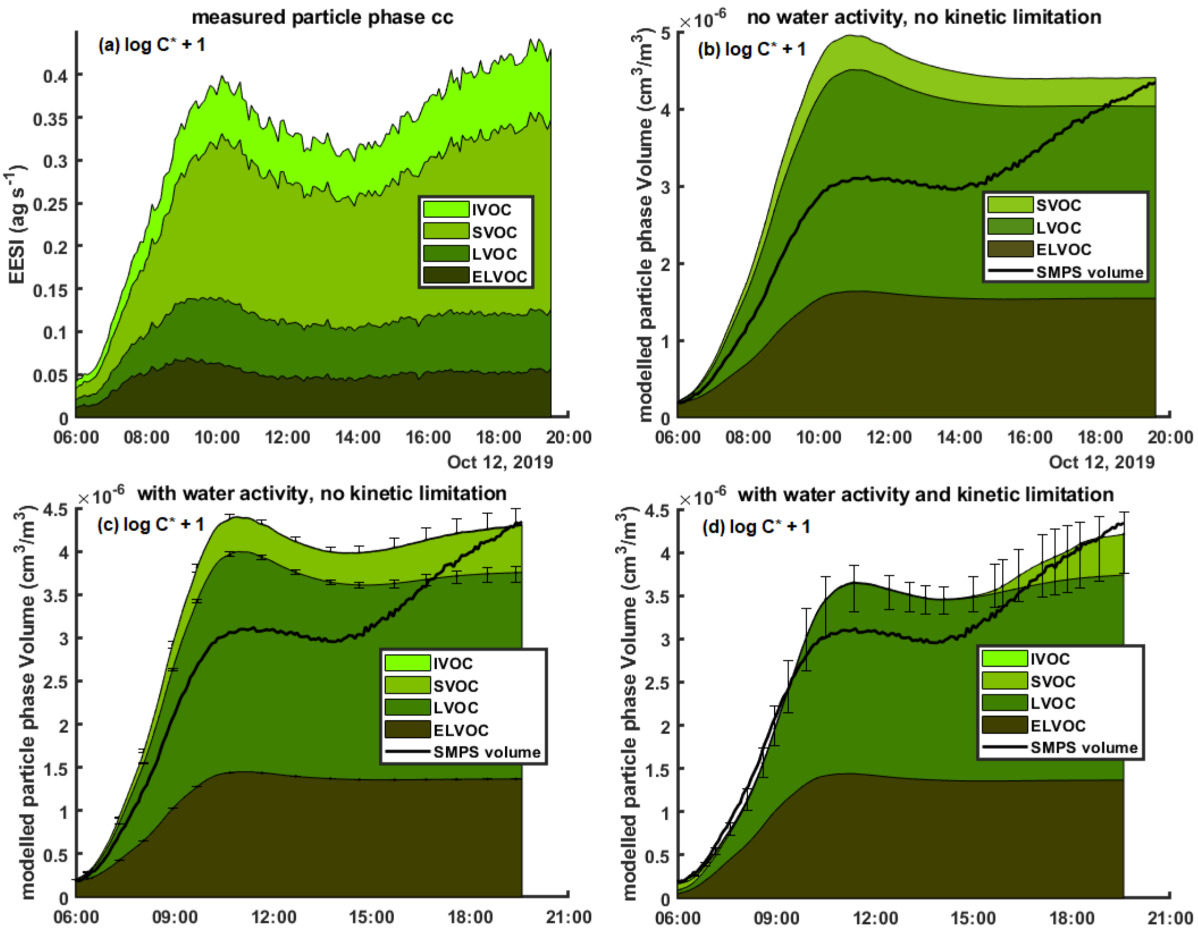

Supplementary Figure 2. Volatility sensitivity test of aerosol growth model with increasing log C^* by 1. Stacked signals of ELVOC, LVOC, SVOC and IVOCs in the particle-phase, as measured by the EESI-TOF **(a)** and volumes measured by the SMPS and predicted by the different models **(b-d)**. Volatility classes are defined at the experimental temperature. The RH is ramped up continuously from 20% at 14:00 to 60% at 19:45. The model in **(b)** takes into account neither water activity nor kinetic limitations to partitioning. The model in **(c)** just takes into account water activity and the model in **(d)** includes both water activity and an easing kinetic limitation with RH. The lower and upper error bars are based on using the growth factor as estimated using Massoli et al. (2010) and Chang et al. (2010), respectively.

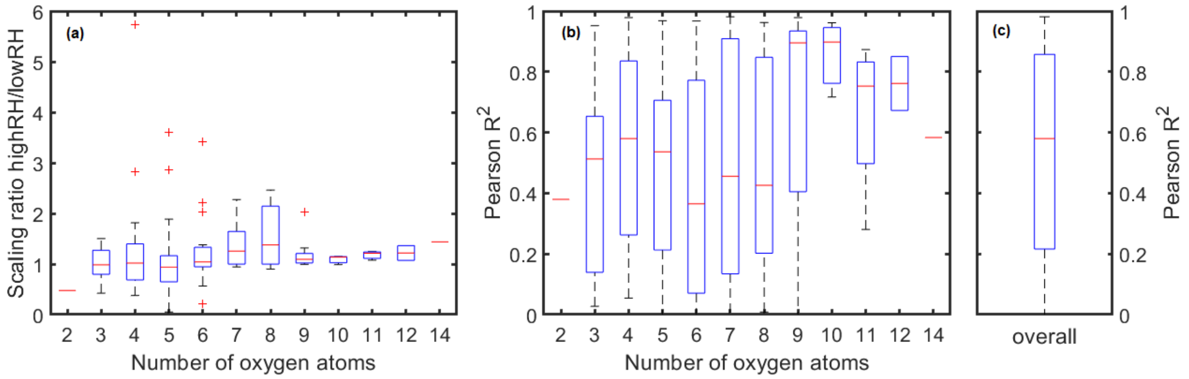

Supplementary Figure 3. Humidity dependence of NH_4^+-CIMS-Orbitrap data, shown as relative change to nitrate-CIMS data at high RH and low RH in **(a)**, binned by number of oxygen atoms. Concentrations were normalized to nitrate-CIMS data at low RH (resulting in a "scaling ratio" of 1.0 for common ions. Panel **(b)** shows correlation coefficients of time series of common ions in the nitrate-CIMS and the NH_4^+-CIMS-Orbitrap, before applying the corrections described in the methods section, while panel **(c)** shows an overall box plot. Data shown here is for Experiment 1, at 243 K.

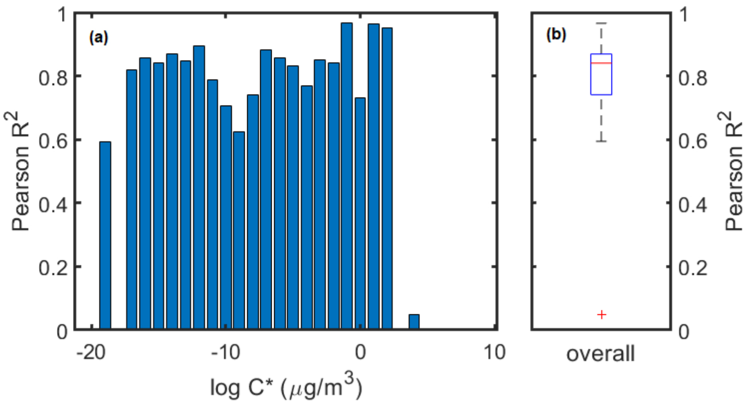

Supplementary Figure 4. Correlation coefficients of time series of concentrations per volatility bin from nitrate-CIMS and NH_4^+-CIMS-Orbitrap data, after applying the corrections described in the methods section. Panel **(a)** shows the data for each volatility bin (log C^*) while panel **(b)** shows an overall box plot. C^* is defined here at 300 K. Data shown here is for Experiment 1, at 243 K.

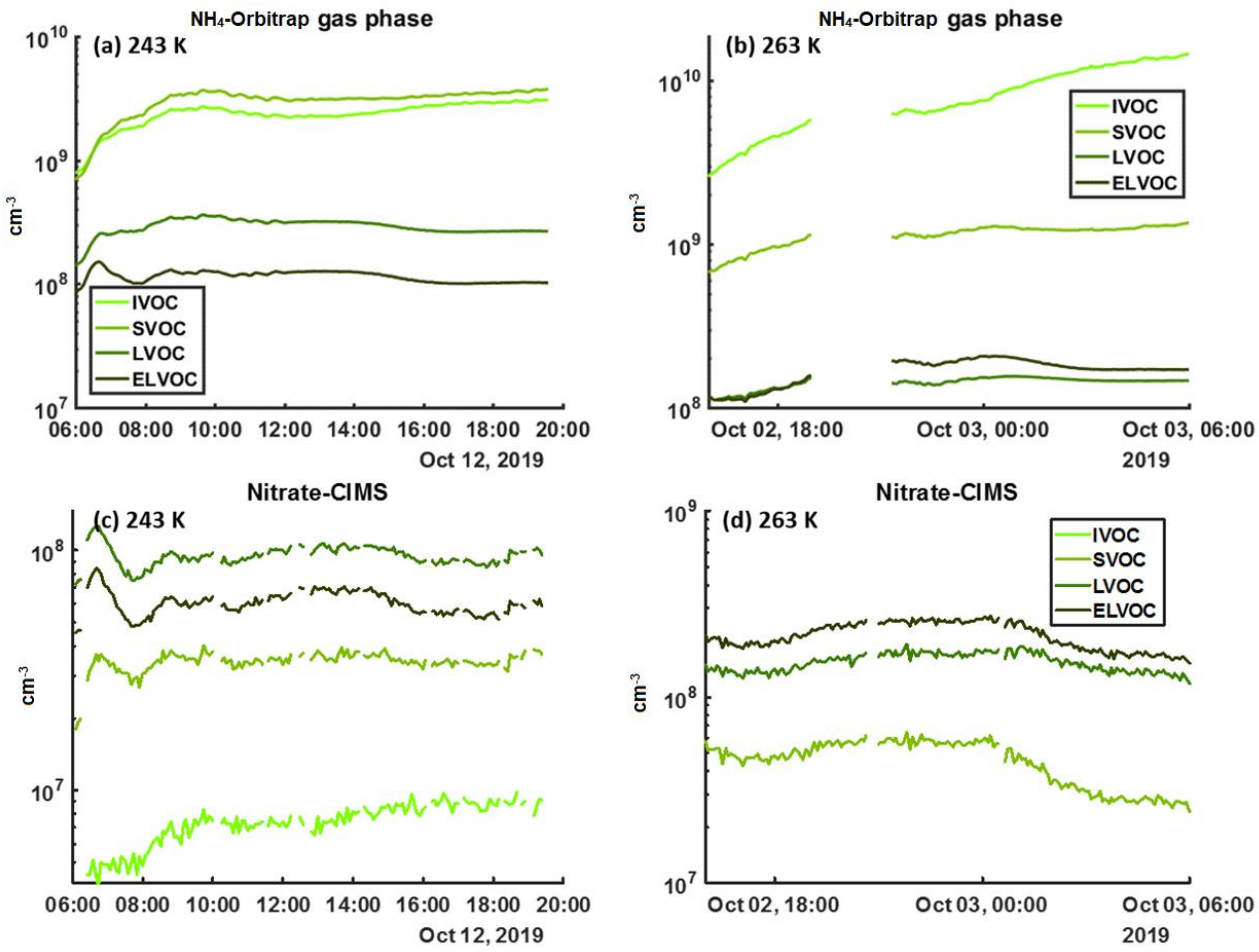

Supplementary Figure 5. Temporal evolution of the gas-phase concentrations of different volatility classes as measured by the NH_4^+-CIMS-Orbitrap (after correction) at 243 K and 263 K in **(a-b)** and by the nitrate-CIMS at the same temperatures in **(c-d)**.

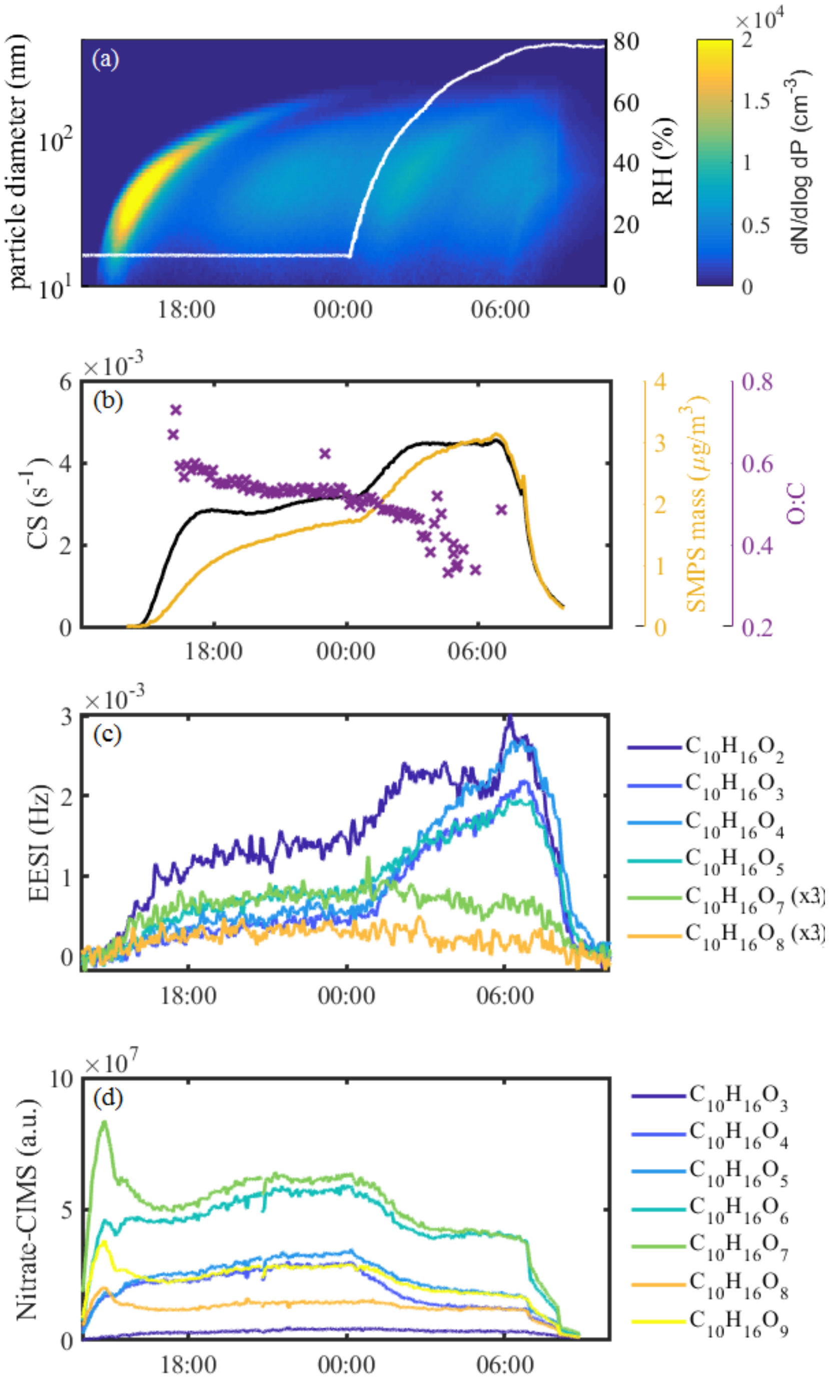

Supplementary Figure 6. Overview of a pure biogenic oxidation experiment where the RH is ramped up. The experiment is carried out at 263 K. **(a)** Particle number size distribution from the SMPS and RH trace overlaid in white. **(b)** Time series of total SMPS mass, condensation sink and O:C ratio. The O:C ratio shown is from the AMS until 03:30, when it is replaced by EESI-TOF data due to an instrumental breakdown. **(c)** Time series of $C_{10}H_{16}O_{2-5,7,8}$ compounds in the particle-phase from the EESI-TOF. $C_{10}H_{16}O_{7,8}$ signals were multiplied by 3 for better visualization. **(d)** Time series of $C_{10}H_{16}O_{3-9}$ compounds in the gas phase from the nitrate-CIMS.

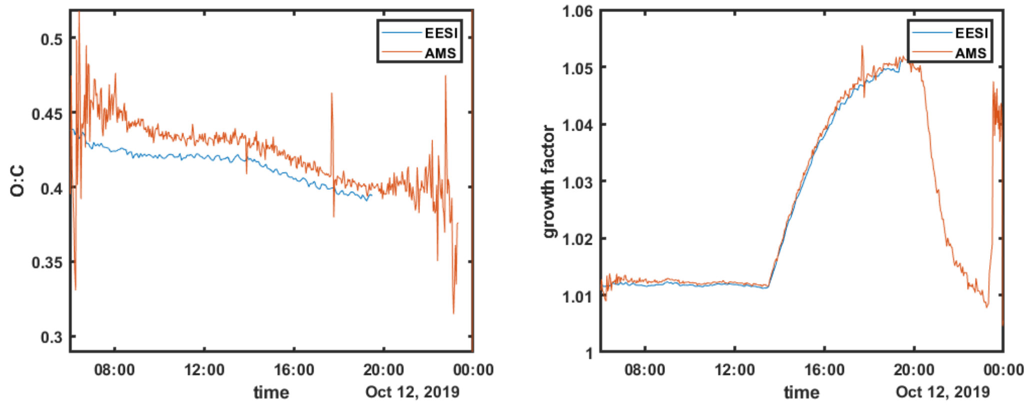

Supplementary Figure 7. Comparison of the O:C **(left panel)** and parameterized growth factors **(right panel)** obtained from the EESI-TOF and AMS for the experiment at 243 K. An average growth factor of the Massoli et al. (2010) and Chang et al. (2010) parameterizations is shown here.

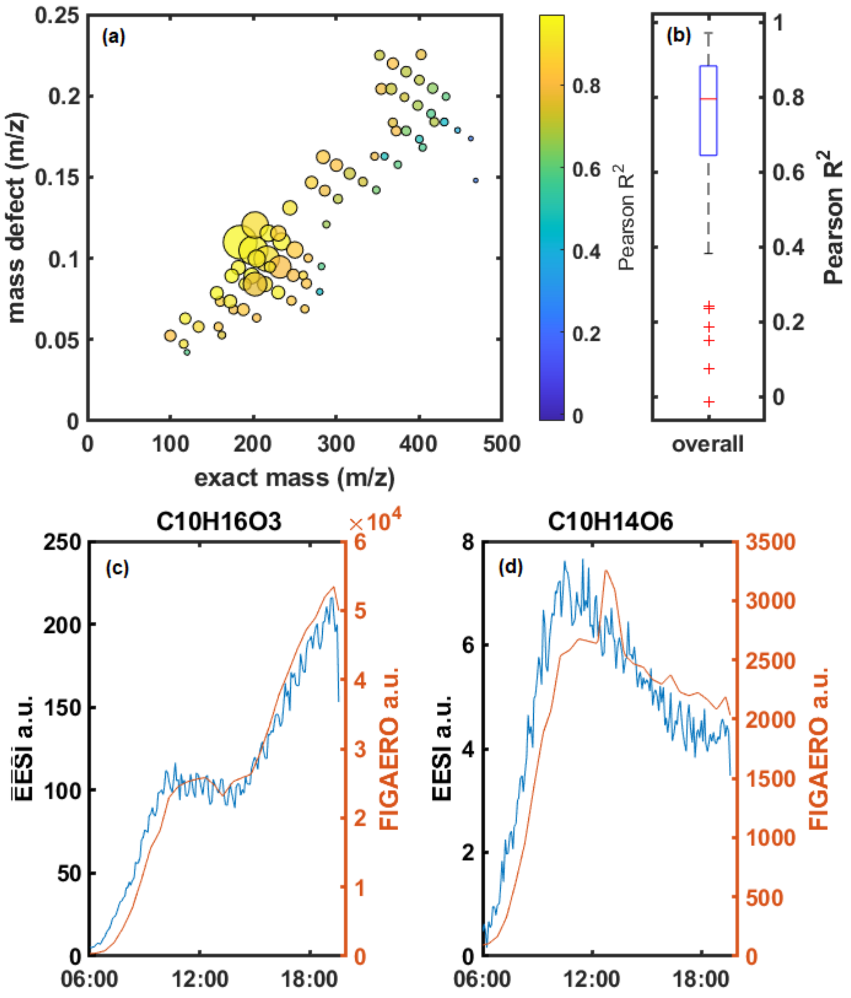

Supplementary Figure 8. Comparison of common ions from the EESI-TOF and FIGAERO-CIMS at 243 K. Panel **(a)** shows a mass defect plot of common ions, colored by their correlation coefficient and sized by the square root of EESI-TOF intensity at high RH conditions. A box plot of the overall R^2 is

given in panel **(b)**. Panels **(c)** and **(d)** show time series of representative ions with different oxygen content ($C_{10}H_{16}O_3$ and $C_{10}H_{14}O_6$, respectively) in the EESI-TOF (left axis) and FIGAERO-CIMS (right axis). The RH is ramped up continuously from 20% at 14:00 to 60% at 19:45.

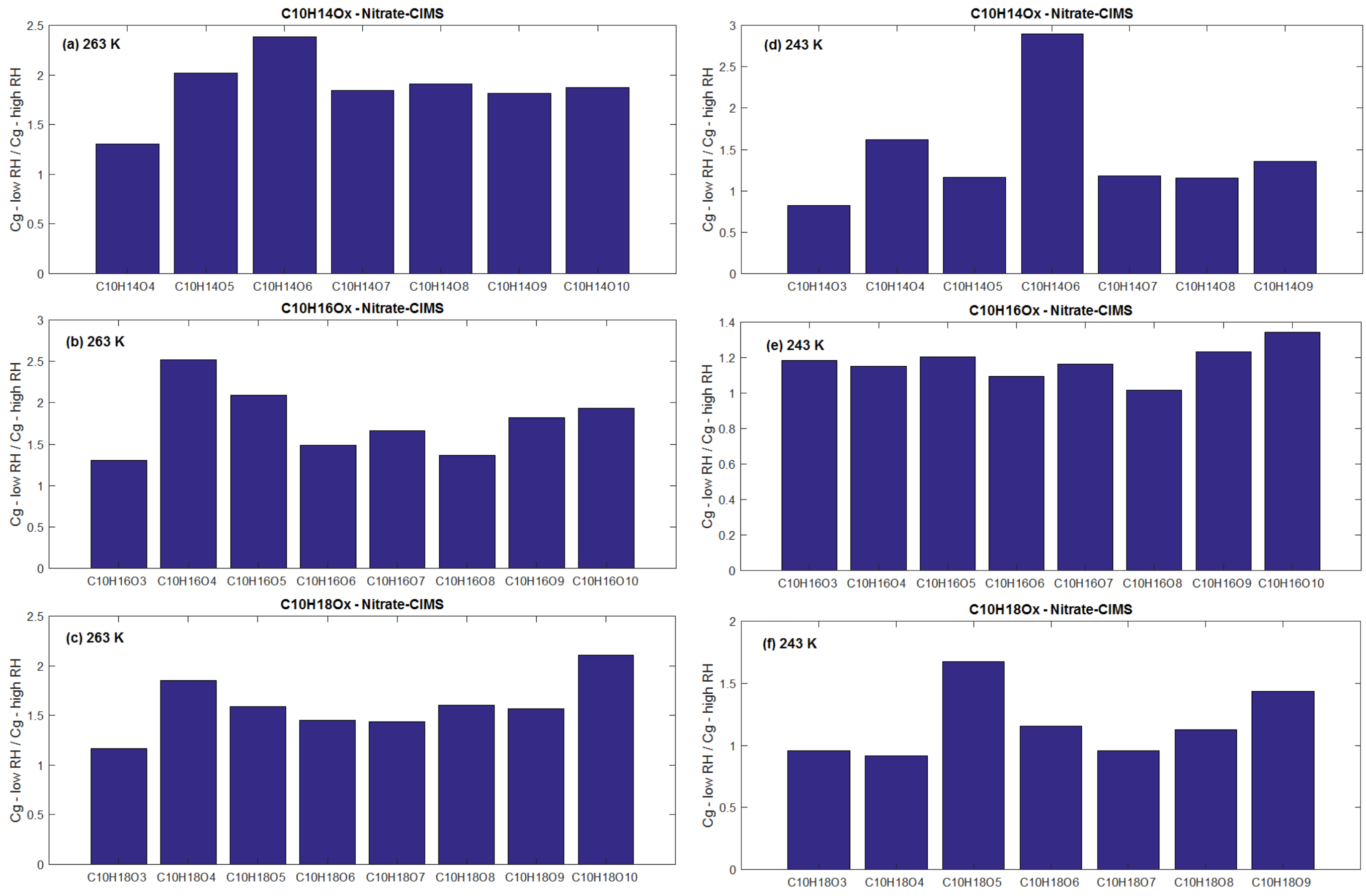

Supplementary Figure 9. Change in gas-phase concentrations of $C_{10}H_{14,16,18}O_x$ species between low RH conditions and high RH conditions, as measured by the nitrate-CIMS. The concentrations of the majority of measured species decrease at high RH as the condensation sink increases. Panels **(a-c)** and **(d-f)** show data from the experiments at 263 and 243 K, respectively.

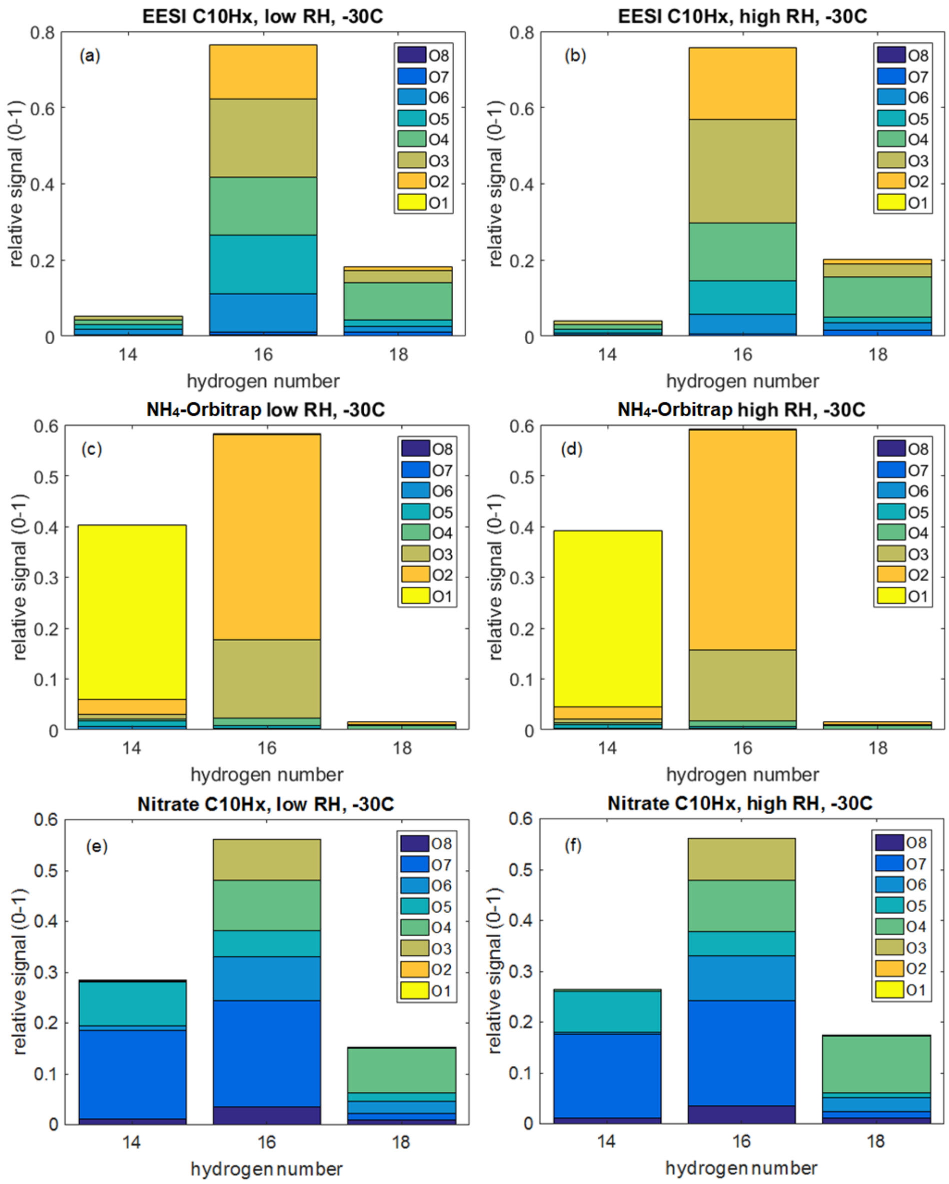

Supplementary Figure 10. Hydrogen number distributions of C_{10} species in the particle and gas phase, at low and high RH, for the experiment at 243 K. Panels **(a)** and **(b)** show data from the particle phase, measured by the EESI-TOF, at low and high RH, respectively. Panels **(c)** and **(d)** show data from the gas phase, measured by the NH_4^+-CI-Orbitrap, at low and high RH, respectively. Panels **(e)** and **(f)** show data from the gas phase, measured by the nitrate-CIMS, at low and high RH, respectively. The difference between the NH_4-Orbitrap and the nitrate-CIMS is due to the low sensitivity of the nitrate-CIMS to weakly oxygenated compounds.

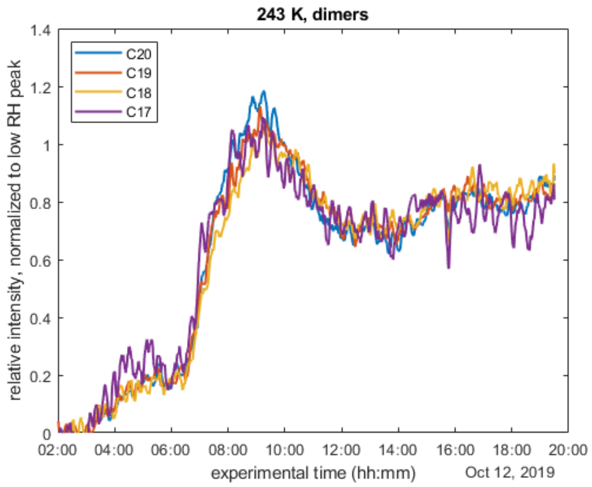

Supplementary Figure 11. Time series of dimer compounds in the particle phase, as measured by the EESI-TOF, grouped by carbon number for the experiments at 243 K. The signals are normalized to the maximum of the signal under low RH conditions. As the dimer signals for the 263 K experiments are noisy due to the dimer mass concentrations nearing the detection limits of the EESI-TOF, it is difficult to draw conclusions and therefore that experiment is not shown.

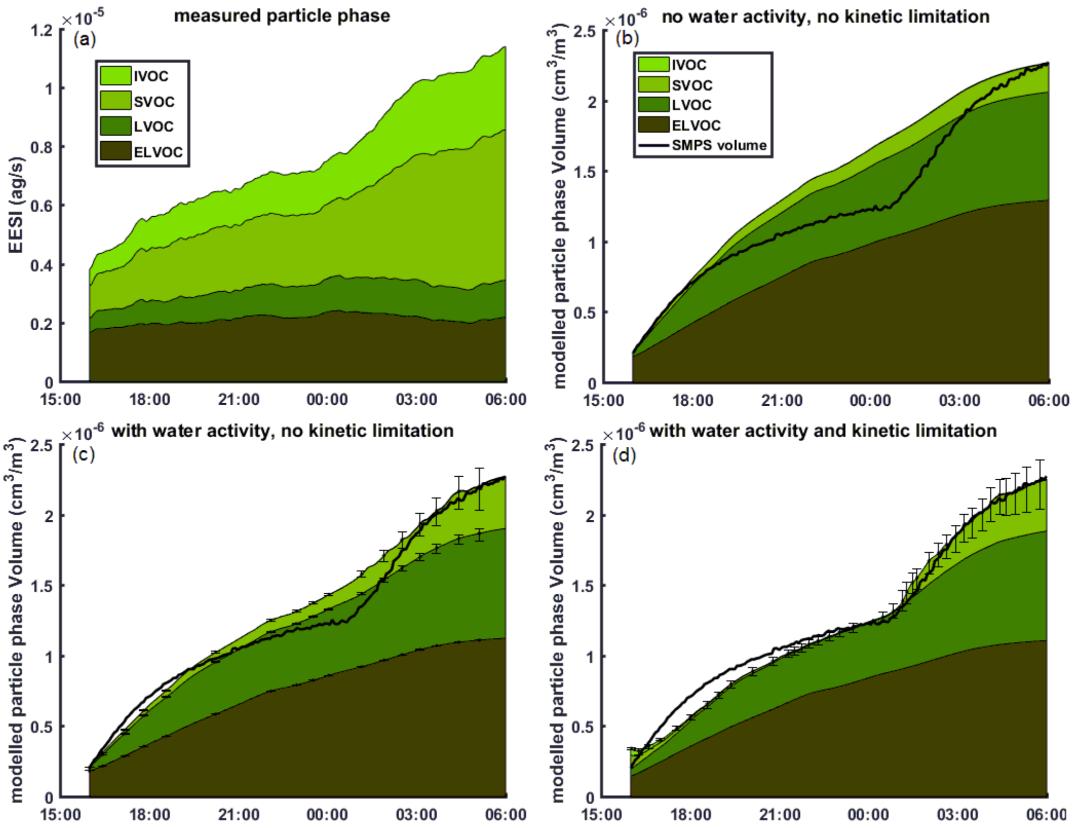

Supplementary Figure 12. Stacked signals of ELVOCs, LVOCs, SVOCs and IVOCs in the particle phase, measured by the EESI-TOF **(a)** and volumes measured by the SMPS and predicted by the different models **(b-d)**, all at 263 K. Volatility classes are defined at the experimental temperature. The RH is ramped up continuously from 10% at 00:30 to 80% at 07:00.The model in **(b)** takes into account neither water activity nor kinetic limitations to partitioning. The model in **(c)** just takes into account water activity and the model in **(d)** includes both water activity and an easing kinetic limitation with RH.

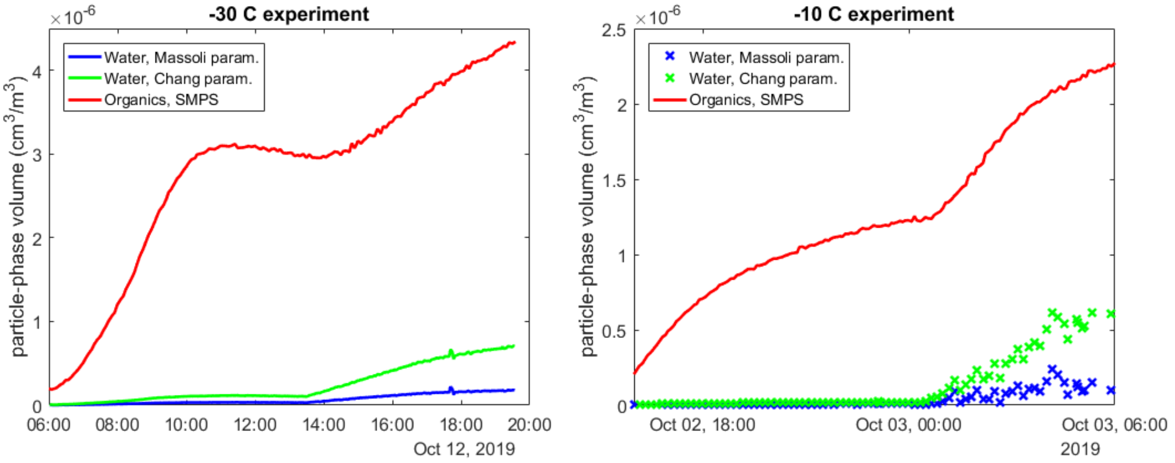

Supplementary Figure 13. Total particle volume as measured by the SMPS (red line), compared to particle-phase water volume, as estimated by Massoli et al. (2010) and Chang et al. (2010), in blue and green, respectively. The panel on the **left** shows data at 243 K while the panel on the **right** shows data at 263 K.

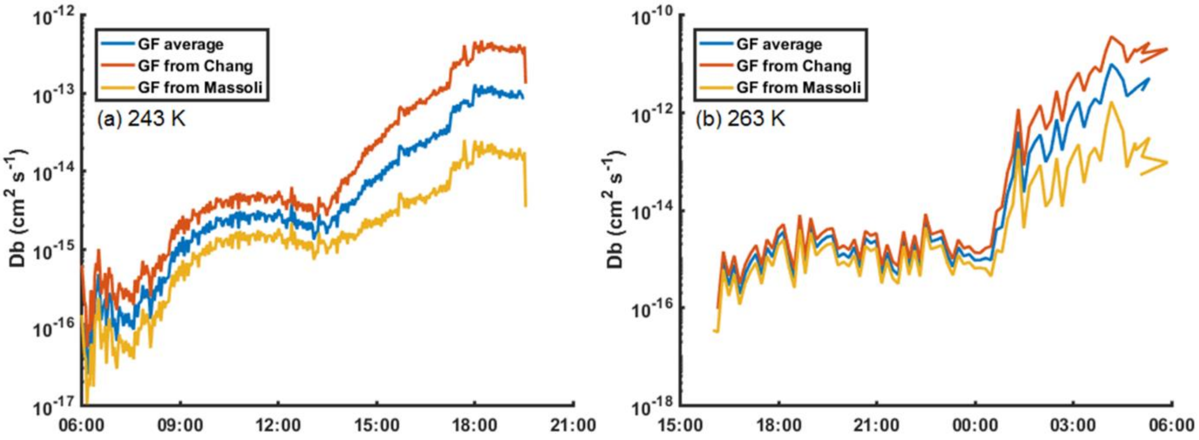

Supplementary Figure 14. Bulk diffusivity values (D_b) used to reach the "best-agreement" model results (in Figures 3c and S5c) at 243 **(a)** and 263 K **(b).** Estimates using the growth factor parametrizations of Massoli et al. (2010), Chang et al. (2010) and an average of the two are given.

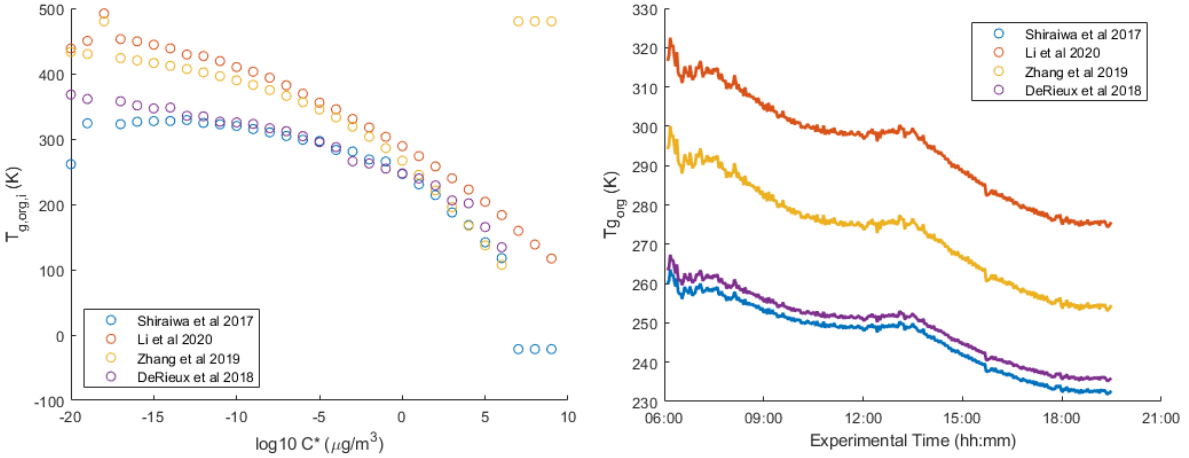

Supplementary Figure 15. Comparison of four different parametrizations for obtaining the glass transition temperature (T_g) from the molecular composition. The **(left panel)** shows the estimated T_g for each volatility bin, whereas the **(right panel)** shows time series of the estimated T_g for the SOA mixture in the experiment at 243 K.

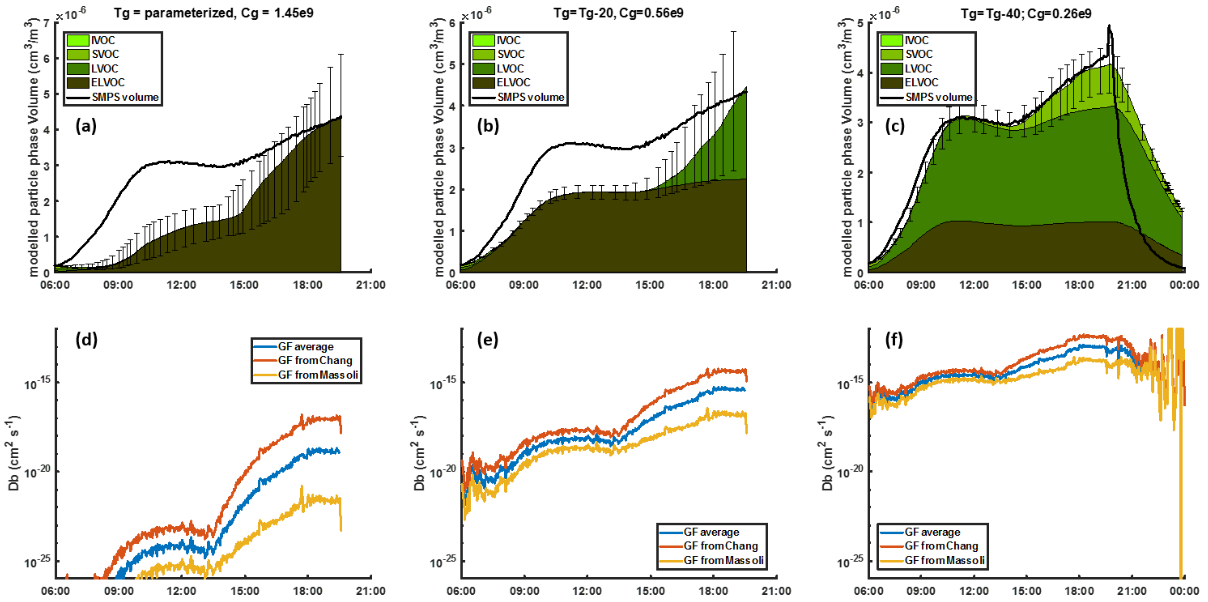

Supplementary Figure 16. Model sensitivity tests to bulk diffusivity (D_b) for the 243 K experiment. Panels **(a), (b)** and **(c)** show the model results when using the parametrized glass transition temperature (T_g) to estimate D_b, T_g *-20* and T_g*-40*, respectively. Gas-phase concentrations were scaled accordingly to match the observed growth at the high RH peak and the scaling factors are stated in the figures as C_g. The obtained D_b values are given in panels **(d), (e)** and **(f)**. Estimates using the growth factor parametrizations of Massoli et al. (2010), Chang et al. (2010) and an average of the two are given.

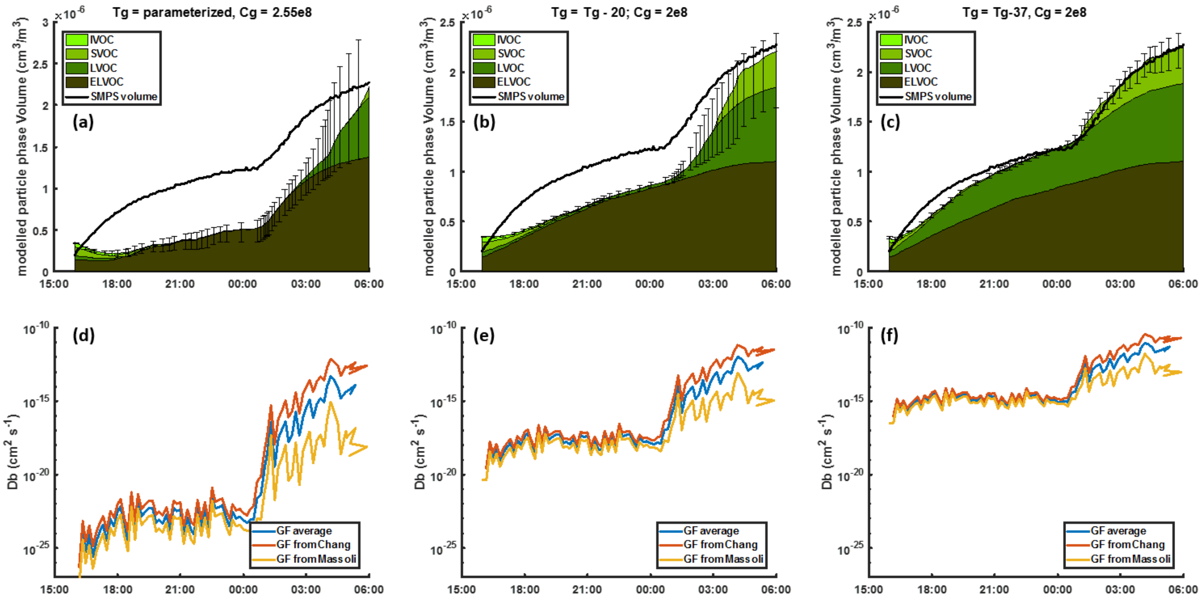

Supplementary Figure 17. Model sensitivity tests to bulk diffusivity (D_b) for the 263 K experiment. Panels **(a), (b)** and **(c)** show the model results when using the parametrized glass transition temperature (T_g) to estimate D_b, T_g *-20* and T_g*-37*, respectively. Gas-phase concentrations were scaled accordingly to match the observed growth at the high RH peak and the scaling factors are stated in the figures as C_g. The obtained D_b values are given in panels **(d), (e)** and **(f)**. Estimates using the growth factor parametrizations of Massoli et al. (2010), Chang et al. (2010) and an average of the two are given.

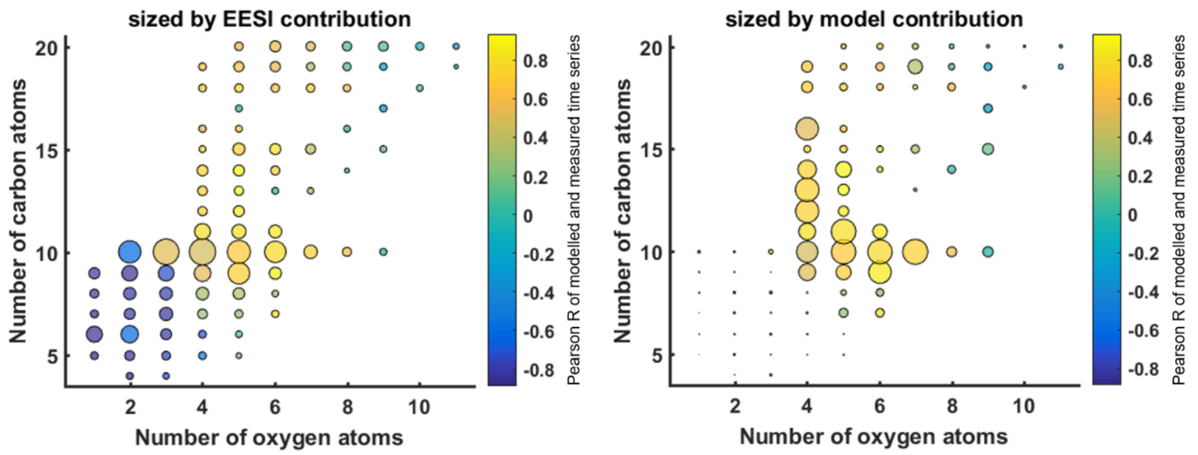

Supplementary Figure 18. Comparison of time series correlations of modelled and measured compounds in the particle phase, on a molecular basis, for the experiment at 243 K. Circles are sized by the square root of their contribution in either the measured EESI-TOF data **(left panel)** or model **(right panel)** at the OA mass peak at high RH. Compounds with same carbon and oxygen atom numbers are summed up, and only species common to both the EESI-TOF and NH_4^+-CIMS-Orbitrap are shown.

References

(1) Atkinson, R.; Aschmann, S. M.; Arey, J.; Shorees, B. Formation of OH Radicals in the Gas Phase Reactions of O3 with a Series of Terpenes. *Journal of Geophysical Research: Atmospheres* **1992**, *97* (D5), 6065–6073. https://doi.org/10.1029/92JD00062.

(2) Chang, R. Y.-W.; Slowik, J. G.; Shantz, N. C.; Vlasenko, A.; Liggio, J.; Sjostedt, S. J.; Leaitch, W. R.; Abbatt, J. P. D. The Hygroscopicity Parameter (κ) of Ambient Organic Aerosol at a Field Site Subject to Biogenic and Anthropogenic Influences: Relationship to Degree of Aerosol Oxidation. *Atmos. Chem. Phys.* **2010**, *10* (11), 5047–5064. https://doi.org/10.5194/acp-10-5047-2010.

(3) DeRieux, W.-S. W.; Li, Y.; Lin, P.; Laskin, J.; Laskin, A.; Bertram, A. K.; Nizkorodov, S. A.; Shiraiwa, M. Predicting the Glass Transition Temperature and Viscosity of Secondary Organic Material Using Molecular Composition. *Atmos. Chem. Phys.* **2018**, *18* (9), 6331–6351. https://doi.org/10.5194/acp-18-6331-2018.

(4) Jenkin, M. E.; Saunders, S. M.; Pilling, M. J. The Tropospheric Degradation of Volatile Organic Compounds: A Protocol for Mechanism Development. *Atmospheric Environment* **1997**, *31* (1), 81–104. https://doi.org/10.1016/S1352-2310(96)00105-7.

(5) Li, Y.; Day, D. A.; Stark, H.; Jimenez, J. L.; Shiraiwa, M. Predictions of the Glass Transition Temperature and Viscosity of Organic Aerosols from Volatility Distributions. *Atmos. Chem. Phys.* **2020**, *20* (13), 8103–8122. https://doi.org/10.5194/acp-20-8103-2020.

(6) Long, B.; Bao, J. L.; Truhlar, D. G. Unimolecular Reaction of Acetone Oxide and Its Reaction with Water in the Atmosphere. *Proceedings of the National Academy of Sciences* **2018**, *115* (24), 6135–6140. https://doi.org/10.1073/pnas.1804453115.

(7) Massoli, P.; Lambe, A. T.; Ahern, A. T.; Williams, L. R.; Ehn, M.; Mikkilä, J.; Canagaratna, M. R.; Brune, W. H.; Onasch, T. B.; Jayne, J. T.; Petäjä, T.; Kulmala, M.; Laaksonen, A.; Kolb, C. E.; Davidovits, P.; Worsnop, D. R. Relationship between Aerosol Oxidation Level and Hygroscopic Properties of Laboratory Generated Secondary Organic Aerosol (SOA) Particles. *Geophysical Research Letters* **2010**, *37* (24). https://doi.org/10.1029/2010GL045258.

(8) Saunders, S. M.; Jenkin, M. E.; Derwent, R. G.; Pilling, M. J. Protocol for the Development of the Master Chemical Mechanism, MCM v3 (Part A): Tropospheric Degradation of Non-Aromatic Volatile Organic Compounds. *Atmospheric Chemistry and Physics* **2003**, *3* (1), 161–180. https://doi.org/10.5194/acp-3-161-2003.

(9) Shiraiwa, M.; Li, Y.; Tsimpidi, A. P.; Karydis, V. A.; Berkemeier, T.; Pandis, S. N.; Lelieveld, J.; Koop, T.; Pöschl, U. Global Distribution of Particle Phase State in Atmospheric Secondary Organic Aerosols. *Nat Commun* **2017**, *8* (1), 15002. https://doi.org/10.1038/ncomms15002.

(10) Stone, D.; Rowley, D. M. Kinetics of the Gas Phase HO_2 Self-Reaction: Effects of Temperature, Pressure, Water and Methanol Vapours. *Phys. Chem. Chem. Phys.* **2005**, *7* (10), 2156–2163. https://doi.org/10.1039/B502673C.

(11) Zhang, Y.; Nichman, L.; Spencer, P.; Jung, J. I.; Lee, A.; Heffernan, B. K.; Gold, A.; Zhang, Z.; Chen, Y.; Canagaratna, M. R.; Jayne, J. T.; Worsnop, D. R.; Onasch, T. B.; Surratt, J. D.; Chandler, D.;

Davidovits, P.; Kolb, C. E. The Cooling Rate- and Volatility-Dependent Glass-Forming Properties of Organic Aerosols Measured by Broadband Dielectric Spectroscopy. *Environ. Sci. Technol.* **2019**, *53* (21), 12366–12378. https://doi.org/10.1021/acs.est.9b03317.
